# Janus-faced *EPHB4*-associated disorders: novel pathogenic variants and unreported intrafamilial overlapping phenotypes

**DOI:** 10.1038/s41436-021-01136-7

**Published:** 2021-04-16

**Authors:** Silvia Martin-Almedina, Kazim Ogmen, Ege Sackey, Dionysios Grigoriadis, Christina Karapouliou, Noeline Nadarajah, Cathrine Ebbing, Jenny Lord, Rhiannon Mellis, Fanny Kortuem, Mary Beth Dinulos, Cassandra Polun, Sherri Bale, Giles Atton, Alexandra Robinson, Hallvard Reigstad, Gunnar Houge, Axel von der Wense, Wolf-Henning Becker, Steve Jeffery, Peter S. Mortimer, Kristiana Gordon, Katherine S. Josephs, Sarah Robart, Mark D. Kilby, Stephanie Vallee, Jerome L. Gorski, Maja Hempel, Siren Berland, Sahar Mansour, Pia Ostergaard

**Affiliations:** 1 Molecular and Clinical Sciences Institute, St George’s University of London, London, UK; 2 Department of Obstetrics and Gynecology, Haukeland University Hospital, Bergen, Norway; 3 Wellcome Sanger Institute, Hinxton, UK; 4 North Thames Genomic Laboratory Hub, Great Ormond Street Hospital for Children NHS Foundation Trust, London, UK; 5 Genetics and Genomic Medicine, UCL Great Ormond Street Institute of Child Health, London, UK; 6 Institute of Human Genetics, University Medical Center Hamburg Eppendorf, Hamburg, Germany; 7 Departments of Pediatrics – Section of Genetics and Child Development, Dartmouth-Hitchcock Medical Center, Lebanon, NH USA; 8 Geisel School of Medicine at Dartmouth College, Hanover, NH USA; 9 Department of Child Health, University of Missouri School of Medicine, Columbia, MO USA; 10 GeneDx, 207 Perry Parkway, Gaithersburg, MD USA; 11 University Hospitals Bristol NHS Foundation Trust, Bristol, United Kingdom; 12 Neonatal intensive care unit, Children’s Department, Haukeland University Hospital, Bergen, Norway; 13 Department of Medical Genetics, Haukeland University Hospital, Bergen, Norway; 14 Department of Neonatology and Paediatric Intensive Care, Altona Children’s Hospital, Hamburg, Germany; 15 Elbe Center for Prenatal Medicine, Hamburg, Germany; 16 Dermatology & Lymphovascular Medicine, St George’s Universities NHS Foundation Trust, London, UK; 17 South West Thames Regional Genetics Service, St George’s NHS Foundation Trust, London, UK; 18 The Institute of Metabolism & Systems Research, College of Medical & Dental Sciences, University of Birmingham, Birmingham, UK; 19 West Midlands Fetal Medicine Centre, Birmingham Women’s & Children’s Foundation Trust, Birmingham, UK

## Abstract

**Purpose:**

Several clinical phenotypes including fetal hydrops, central conducting lymphatic anomaly or capillary malformations with arteriovenous malformations 2 (CM-AVM2) have been associated with *EPHB4* (Ephrin type B receptor 4) variants, demanding new approaches for deciphering pathogenesis of novel variants of uncertain significance (VUS) identified in *EPHB4*, and for the identification of differentiated disease mechanisms at the molecular level.

**Methods:**

Ten index cases with various phenotypes, either fetal hydrops, CM-AVM2, or peripheral lower limb lymphedema, whose distinct clinical phenotypes are described in detail in this study, presented with a variant in *EPHB4*. In vitro functional studies were performed to confirm pathogenicity.

**Results:**

Pathogenicity was demonstrated for six of the seven novel *EPHB4* VUS investigated. A heterogeneity of molecular disease mechanisms was identified, from loss of protein production or aberrant subcellular localization to total reduction of the phosphorylation capability of the receptor. There was some phenotype–genotype correlation; however, previously unreported intrafamilial overlapping phenotypes such as lymphatic-related fetal hydrops (LRFH) and CM-AVM2 in the same family were observed.

**Conclusion:**

This study highlights the usefulness of protein expression and subcellular localization studies to predict *EPHB4* variant pathogenesis. Our accurate clinical phenotyping expands our interpretation of the Janus-faced spectrum of *EPHB4*-related disorders, introducing the discovery of cases with overlapping phenotypes.

## INTRODUCTION

The *EPHB4* gene encodes a receptor tyrosine kinase protein, EPHB4, that binds to its ligand EphrinB2, to initiate complex contact-dependent bidirectional signaling cascades, controlling cellular fate during embryonic angiogenesis and essential cellular processes such as adhesion, migration, and proliferation, in both blood and lymphatic endothelial cells.^1^

We previously reported that monoallelic missense variants in the intracellular tyrosine kinase domain of *EPHB4* cause a form of in utero primary lymphatic anomaly coined lymphatic-related fetal hydrops with/without atrial septal defect (LRFH) (OMIM 617300).^2^ The study included two families (GLD_UK_ and GLD_NOR_) with a primarily lymphatic and venous phenotype, but with several family members presenting with fetal hydrops and/or atrial septal defects (ASD). Supporting evidence from mouse models lacking Ephb4 expression during specific stages of development, or genetically modified to alter only the Ephb4 dependent forward signaling, show edema, blood filled lymphatic vessels with defective collector valves, and lymphovenous valves,^2,3^ which suggested the EPHB4-associated hydrops in the two families was caused by a lymphatic-related fault. Li et al. identified an in-frame insertion in EPHB4 in a family with a history of fetal hydrops and lymphovenous dysfunction characterized by edema of the lower extremities, venous stasis, and variable chylous effusions.^4^ This work confirmed the important role of EPHB4 in the function and development of the lymphatic system and the association of *EPHB4* variants with fetal hydrops of lymphovenous origin.

Other research groups have reported that loss-of-function, monoallelic variants in the same gene can cause various vascular pathologies. Under the umbrella of capillary malformation–arteriovenous malformation 2 (CM-AVM2) (OMIM 618196) are included several vascular pathologies such as isolated multifocal capillary malformations, telangiectasia, or high flow complex arteriovenous malformations.^5–8^ Some patients with telangiectases consistent with hereditary hemorrhagic telangiectasia (HHT) but negative for the classical HHT-associated genes have been positively screened for *EPHB4* variants suggesting that the spectrum of the CM-AVM2 phenotype should be expanded.^9^ *EPHB4* variants have been detected in patients with cutaneous telangiectases, initially suspected to have HHT, but negative for variants in the classical HHT-associated genes. Upon review of these cases regarding specific location, number, and appearance of the telangiectases, they were noted to be distinct from those seen in patients with HHT. The cutaneous telangiectases in these patients with an *EPHB4* variant had an earlier onset (childhood) and were often innumerable in a given location. Some of the larger lesions (labeled as capillary malformations [CMs]) were “haloed” and occurred in locations (trunk and extremities) not typical of HHT. These CMs probably represent larger dermal AVMs.

The increasing number of *EPHB4* variants of uncertain significance (VUS) identified through exome sequencing demands rapid and effective tools to confirm pathogenicity to enable an accurate genetic diagnosis. No clear phenotype–genotype correlation associated with *EPHB4* variants has yet been identified and our understanding of the disease mechanism of the known causal variants that could assist in clinical decision making is limited.

In this study we aimed to functionally investigate seven novel *EPHB4* VUS (from eight unreported index cases) associated with a disease phenotype to predict their pathogenicity and attempted to unravel the molecular disease mechanisms that could explain the variable phenotypes. For comparison, three variants from previously reported cases and three control variants selected from gnomAD were also included in this investigation. We demonstrated the pathogenic effect of six of the seven variants, showing a variety of EPHB4-related disease mechanisms at a molecular level.

We present an increasing number of variable or overlapping phenotypes associated with *EPHB4* variants. These include a dominant form of primary lymphedema without fetal hydrops, ASD, or capillary malformation, and an intrafamilial overlapping phenotype with capillary malformation (telangiectasia) and ASD, with other members of the family presenting with fetal hydrops but no telangiectasia, i.e., a dual (Janus-faced) phenotype. This highlights that a detailed clinical evaluation of the affected families and continuous follow-up are critical. Functional validation of the causative variants was undertaken, and various disease mechanisms put forward. This knowledge will help in understanding any phenotype–genotype correlations, and aid clinical practice.

## MATERIALS AND METHODS

### Patient selection, *EPHB4* variant detection, and lymphoscintigraphy

Ten index cases and affected family members with variants in *EPHB4* were included in the study. Two were previously described,^2^ and eight were novel, unreported referrals. Of those, five cases were direct referrals from clinicians, two were identified through the Prenatal Assessment of Genomes and Exomes (PAGE) study,^10^ and one was identified through GeneMatcher.^11^ See Supplementary Information, Supplementary Table 1, and Supplementary Figs. 1–3 for detailed clinical information of the recruited cases. *EPHB4* variants were detected by next-generation sequencing (NGS) in the respective molecular genetics services and confirmed by Sanger sequencing.

Patients from the GLD_UK_, GLD_NOR_, and PL1 families underwent lower limb lymphoscintigraphy, which was performed according to standard local procedure by injecting radioactive isotope (technetium-99m-nanocoll) into the web spaces between the toes.^12,13^ Images were taken at 15 minutes and 2 hours postinjection with a gamma camera. Quantification figures 2 hours postinjection were calculated, where possible, as percentage (%) of tracer retention within right and left foot, and tracer uptake in the ilioinguinal nodes.

### In silico analysis

The relative genomic and protein positions of EPHB4 reported here correspond to the transcript EPHB4–001 (RefSeq: NM_004444, Ensembl: ENST00000358173.3) and P54760 Uniprot protein accession ID. The reported genomic coordinates refer to the GRCh38/hg38 human genome reference. Putative changes in the gene structure and/or amino acid sequence caused by the reported variants were retrieved from the University of California–Santa Cruz (UCSC) Refgene database.^14^ Allele frequencies (AF) were checked in gnomAD databases.^15^ Pathogenicity was predicted by the Combined Annotation Dependent Depletion (CADD) tool,^16^ MutationTaster,^17^ and PolyPhen-2.^18^ The protein model of EPHB4 was generated in R using the Bioconductor trackViewer package.^19^ Multiple sequence alignments of EPHB4 protein from different species were performed using the T-Coffee tool.^20^

### Site-directed mutagenesis of EPHB4 constructs

Fifty nanograms of the EPHB4 mammalian expression vector Myc-DDK-tagged pCMV6-Entry-EPHB4 (RC208559, OriGene) was used as template with the QuikChange II XL Site-Directed Mutagenesis Kit (Agilent). All primers (Supplementary Table 2) were designed with QuikChange Primer Design (Agilent) and all constructs verified by Sanger sequencing.

### Cell culture, transfection, and activation of EPHB4 receptor

Human dermal lymphatic endothelial cells (LECs) (C-12217, PromoCell) were cultured in supplemented endothelial growth medium MV2 (C-22022, PromoCell) containing 50 ng/ml VEGF-C (9199-VC-025, Bio-Techne). The cells are routinely analyzed by flow cytometric analysis and immunofluorescent staining: >95% of the cells are CD31 positive and podoplanin positive. All experiments with LECs were repeated *n* ≥ 3 with cells isolated from independent single donors.

The day before transfection 2×10^5^ cells/well were seeded in fibronectin-coated (F1141, Sigma-Aldrich) 6-well plates. They were then transfected with 2 µg of the EPHB4 constructs and Viromer Yellow transfection reagent (VY-01LB-01, Lipocalyx) or 1 µg of the EPHB4 constructs and Lipofectamine 3000 transfection reagent (Life Technologies). Ligand activation of EPHB4 receptor was performed as previously described.^2^

### Immunoprecipitation and western blot

Twenty-four hours post-transfection LECs were harvested in lysis buffer and immunoprecipitation performed as previously described.^2^ Immunoprecipitates were separated by sodium dodecyl sulfate–polyacrylamide gel electrophoresis (SDS-PAGE) and transferred to Immobilon-FL PVDF membranes (Millipore). Membranes were blocked with TBS blocking solution (Odyssey Blocking Buffer diluted 1:1 in TBS, LI-COR Biosciences) and incubated with goat antihuman EPHB4 (AF3038, Bio-Techne) and mouse antiphosphotyrosine (clone 4G10, 05–321, Millipore) antibodies diluted in TBS blocking solution containing 0.2% Tween 20 (Sigma-Aldrich). After incubation with IRDye 680RD donkey antigoat IgG (925–68074, LI-COR Biosciences) and IRDye 800CW donkey antimouse IgG (925–32212, LI-COR Biosciences) antibodies diluted in TBS blocking solution containing 0.2% Tween 20 (Sigma-Aldrich) and 0.01% SDS (Sigma-Aldrich), membranes were scanned with the Odyssey infrared imaging system (LI-COR Biosciences).

For the detection of EPHB4 expression levels, after transfection, cell lysates were subjected to western blotting as above but instead, EPHB4 expression was detected with C-terminal specific mouse anti-DDK (clone 4C5, TA50011, OriGene), and mouse anti-GAPDH (clone 6C5, MAB374, Millipore) was used as an internal control. Uncropped western blots are shown in Supplementary Figure 7.

### Immunofluorescence and microscopy

Human dermal LECs were seeded on fibronectin-coated glass coverslips prior to lipofectamine-mediated transfection with EPHB4 variants. After 24 hours transfection, cells were fixed with 3% paraformaldehyde (PFA), then permeabilized and quenched with 0.2% saponin/50 mM NH_4_CI. Mouse anti-DDK and goat anti-VE-Cadherin (sc-6458, Santa Cruz Biotechnology) were used as primary antibodies. Alexafluor488 antimouse (A32766, Invitrogen) and Alexafluor555 antigoat (A32816, Invitrogen) were used as fluorophore-conjugated secondary antibodies. All antibody dilutions were prepared in PBS containing 0.2% (w/v) fish skin gelatin, 0.02% (w/v) saponin, and 0.02% (w/v) NaN_3_. DAPI (Sigma-Aldrich) was used for nuclei detection and VECTASHIELD® (Vector Laboratories) was used as mounting medium. Images were taken at 20× magnification with the EVOS™ M5000 imaging system (Thermo Fisher Scientific, Waltham, MA, USA) and analyzed in GIMP (www.gimp.org).

## RESULTS

### Novel *EPHB4* variants related to fetal hydrops of unknown etiology

**Fig. 1:**
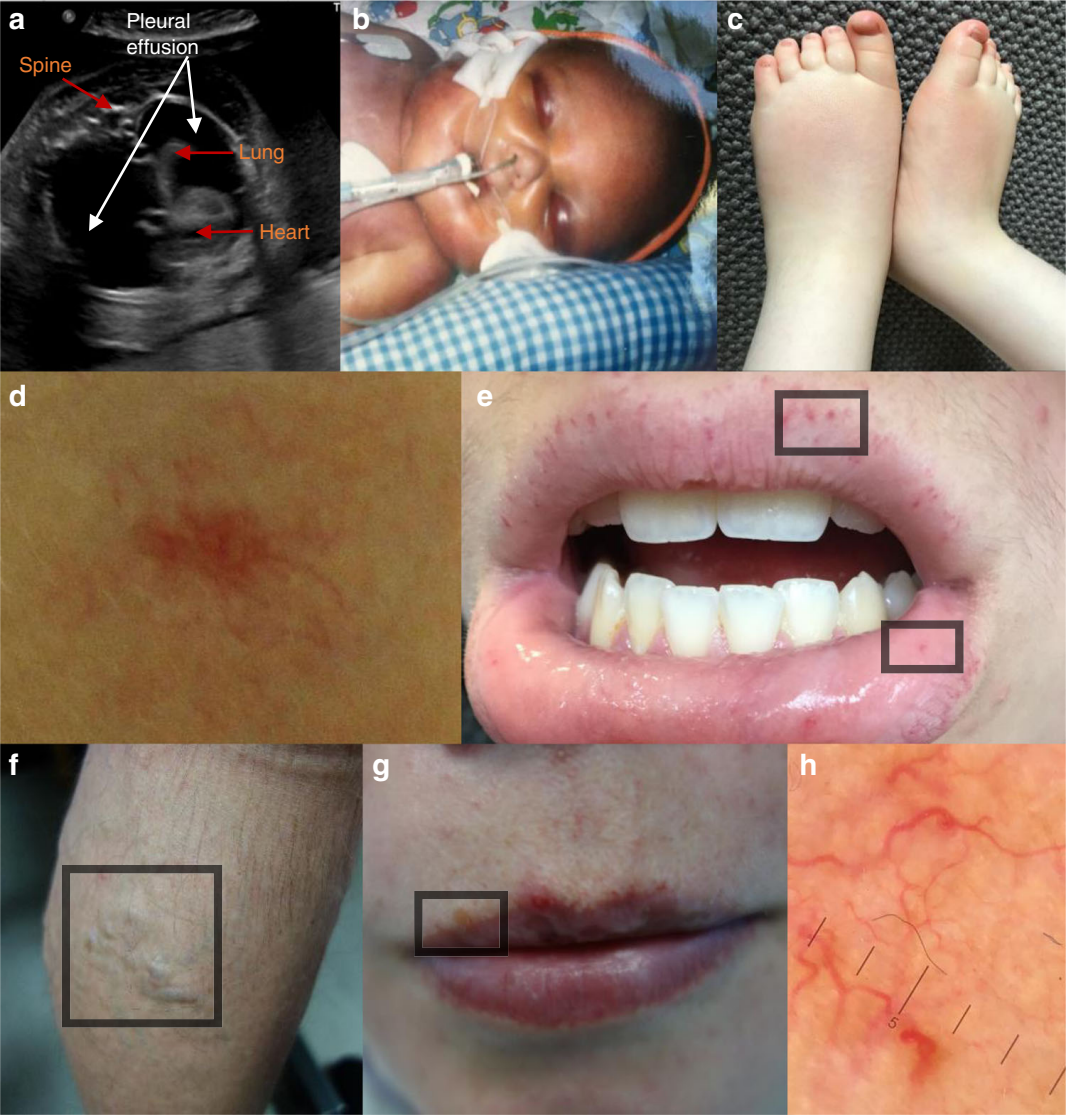
Clinical findings in individuals with *EPHB4* variants. (**a**) Antenatal ultrasound scan (transverse plane) demonstrating bilateral pleural effusions at gestational week 30 + 4 in FH2:II.1. (**b**) Baby in the neonatal period with fetal hydrops (GLD_UK_:II.6). (**c**) Persistent peripheral lymphedema in the feet of FH5:II.2 at age 4 years. (**d**) Capillary malformation in the midline of the neck in VA1:II.2. (**e**) Multiple telangiectasia along the vermilion border of the upper lip and the mucous membrane of the lower lip in VA2:II.1 (inside boxed areas). (**f**) Early onset and extensive lower limb varicose veins in GLD_UK_:I.2. (**g**) GLD_UK_:II.4 with multiple telangiectasia with a propensity for the vermilion border of the lips (inside boxed area). (**h**) Dermatoscopic image of telangiectasia on the left cheek confirming the presence of dilated linear and branching capillary vessels in GLD_UK_:II.4.

Since the first report of two pathogenic *EPHB4* missense variants associated with LRFH,^2^ four additional cases of fetal hydrops (FH1:II.2, FH2:II.1, FH4:II.1, and FH5:II.2) and one case presenting with bilateral pleural effusions (FH3:II.7) with variants in *EPHB4* have come to our attention (Fig. 1a–c). Including other family members, who carried an *EPHB4* variant, a total of seven individuals are reported all presenting with lymphovenous problems and/or congenital heart defects (CHD); for full clinical details and pedigrees see the Supplementary Information, Supplementary Table 1, and Supplementary Figs. 1, 2.

The two pathogenic variants reported by Martin-Almedina et al. are located in the intracellular tyrosine kinase domain.^2^ Of the five new referrals, four carried missense *EPHB4* VUS also located in the same region (Supplementary Figure 4) and conservation analysis revealed that all the variants alter highly conserved residues in the tyrosine kinase domain of EPHB4 (Supplementary Figure 5A). The p.R744H variant was reported in two unrelated cases (FH1:II.2 and FH2:II.1) (Table 1) and is located in a highly conserved region in close proximity to the catalytic loop HRD (His-Arg-Asp), also strongly conserved in most protein kinases (Supplementary Figure 5B) and predicted to control the active conformation of the kinase domain.^21,22^ FH4:II.1 carried an indel (c.760_761insC) predicted to cause a frameshift resulting in the premature truncation of the EPHB4 protein (p.S254Tfs*10), which would lack several functional domains including the tyrosine kinase domain (Supplementary Figure 4). None of the variants were reported in gnomAD and in silico analysis predicts them to be nonfunctional (Table 1).

**Table 1:** Functional annotation of the thirteen *EPHB4* variants investigated in this study.

Genomic coordinates, nucleotide and protein changes, predicted pathogenicity, and population allele frequencies are summarized. All previously reported *EPHB4* variants for the GLD_UK_, GLD_NOR_, and CM-AVM2 cases and *EPHB4* variants of uncertain significance (VUS) for the FH1–FH5, VA1–VA2, and PL1 cases are either not reported in gnomAD databases or their allele frequency is infinitesimal, supporting the argument that they are extremely rare in the general population, while they are all reported to be pathogenic by all three prediction tools used (CADD, PolyPhen-2, MutationTaster). The three *EPHB4* variants used as controls (SNP) in this study are reported as rare variants (AF < 0.01) in gnomAD databases. Two of them are found in homozygotes and/or are predicted as pathogenic. GLD_UK_ and GLD_NOR_ published in Martin-Almedina et al.^2^ and CM-AVM2 in Yu et al.^5^

*AF* allele frequency, *CM-AVM* capillary malformation–arteriovenous malformation, *FH* fetal hydrops, *FND* fibronectin domain, *LBD* ligand binding domain, *PL* primary lymphedema, *SNP* single-nucleotide polymorphism (likely benign variant), *TKD* tyrosine kinase domain, *VA* vascular anomalies.

### Novel *EPHB4* variants related to a vascular anomaly phenotype

The findings from Amyere et al.^6^ and Wooderchak-Donahue et al.^9^ led to the identification in our genetics clinic of two index cases presenting with familial telangiectasia with novel variants in *EPHB4*. There were five affected individuals from two families (VA1 and VA2) who presented with HHT-like features (Fig. 1d, e). For the full clinical details see Supplementary Information, Supplementary Table 1, and Supplementary Figure 3. Affected family members of VA1 carry a stop-gain, p.Q711X, leading to a premature stop codon in the tyrosine kinase domain (Supplementary Figure 4), and VA2 family members a missense variant, p.T110P, in the extracellular ligand binding domain in a highly conserved residue (Supplementary Figures 4, 5A). None of the variants are in gnomAD and in silico analysis predicts them to be nonfunctional (Table 1).

### Novel *EPHB4* variant related to isolated persistent peripheral primary lymphedema

**Fig. 2:**
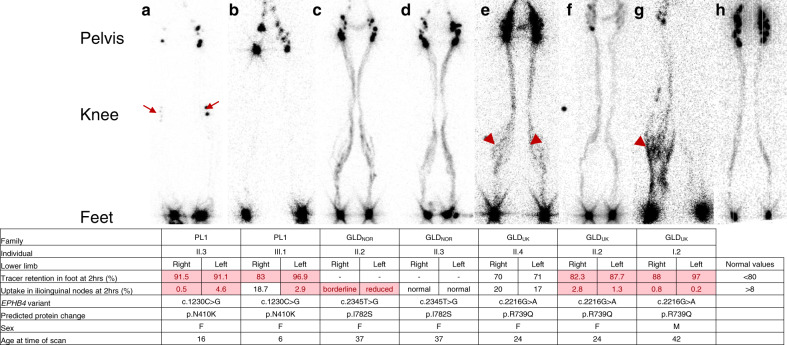
Imaging of the lymphatic system in individuals with *EPHB4* variants. Anterior view of lower limb lymphoscintigraphy 2 hours after injection. Quantification figures 2 hours postinjection are given where available. Values for an individual with a normal lymphatic system are given on the right. Any values deviating from the normal values, indicating abnormal lymphatic drainage, are highlighted in red. Genetic variants and predicted protein changes are also shown. Refer to the Supplementary Information for a detailed description of the lymph scans. (**a**) PL1:II.3 demonstrated abnormal lymphatic drainage in both legs with evidence of bilateral deep rerouting via the popliteal lymph nodes (arrows). (**b**) PL1:III.1 has abnormal drainage, possibly due to deep rerouting but the popliteal lymph nodes are not visible in this scan. (**c**) GLD_NOR_:II.2 has multiple tortuous lymphatic tracts in the lower limbs. Superficial rerouting of tracer is apparent in the calves. (**d**) GLD_NOR_:II.3 has tortuous lower limb lymphatic tracts and superficial rerouting of tracer is present around the ankles and calves. (**e**) The lower limb lymphatic tracts in GLD_UK_:II.4 are tortuous and superficial rerouting is seen within the calves (dark shading, arrowheads). (**f**) GLD_UK_:II.2 has symmetrical impairment of lymphatic drainage within both lower limbs. The main lymphatic tracts are tortuous. (**g**) GLD_UK_:I.2 has superficial rerouting of tracer in the right leg (arrowhead) and deep rerouting via the right popliteal lymph nodes. Lymphatic tracts in the right limb are tortuous. There is markedly reduced lymphatic transport in the left limb with no visible uptake of tracer in the left inguinal lymph nodes. (**h**) Unaffected subject with symmetrical transport of radionuclide tracer from injection sites in the feet up to the inguinal lymph nodes via main lymphatic vessels. The black dot in (**f**) is the orientation marker. F female, M male.

After the screening of a patient (PL1:II.3) with primary lymphedema on the primary lymphedema gene panel through South West Thames Regional Genetic Services, a VUS in *EPHB4* was identified. All three affected family members in PL1 carried this *EPHB4* variant and the main clinical feature was early onset (around age of 2 years) swelling of the lower limbs. There was no history of fetal hydrops, CHD, CM, AVM, or telangiectases. Lymphoscintigraphy imaging was consistent with primary lymphedema (Fig. 2a, b). The full clinical details and the pedigree are presented in the Supplementary Information, Supplementary Table 1, and Supplementary Figure 3. The *EPHB4* VUS identified, p.N410K, is located in the extracellular fibronectin domain (Supplementary Figure 4) in a highly conserved residue (Supplementary Figure 5A), is not reported in gnomAD, and in silico analysis predicts it to be nonfunctional (Table 1).

### Update on clinical findings in previously published *EPHB4*-associated LRFH cases

Since the first report in 2016,^2^ the two families, GLD_UK_ and GLD_NOR_, have been periodically evaluated at the respective specialist centers. Despite no apparent clinical signs of persistent peripheral lymphedema, three individuals underwent lymphoscintigraphy imaging, which confirmed abnormal lower limb lymphatic function (Fig. 2c, d, f). Lymphoscintigraphy in the GLD_NOR_ and GLD_UK_ cases shared features of abnormal lymphatic drainage via tortuous tracts with superficial rerouting similar to previous findings in the GLD_UK_ family (Fig. 2e, g).^2^ Features suggestive of venous hypertension contributing to impaired lymphatic function were also observed. This is in keeping with the observation of often extensive varicose veins from a young age in all five adults investigated in the two families (Fig. 1f). Updated clinical details are given in the Supplementary Information, Supplementary Table 1, and Supplementary Figure 1.

At the time of the original publication, none of the individuals in the GLD_UK_ and GLD_NOR_ families were assessed for telangiectases or capillary malformations. At a recent visit, GLD_UK_:III.2 reported repeated nose bleeds and on further inspection several telangiectases were identified around his mouth. It was also noticed that his mother (GLD_UK_:II.4) had multiple, diffuse telangiectases around the mouth and on her hands (Fig. 1g, h). Both of these individuals have ASDs (III.2 had multiple ASDs) and neither were hydropic or had pleural effusions unlike other affected family members. Brain and spine magnetic resonance images (MRIs) were entirely normal, no AVMs were identified, and there were no capillary malformations. No pathogenic variants in the HHT genes (*ENG*, *ACVRL1*, *GDF2*, and *SMAD4*) or *RASA1* were identified in the exome data of GLD_UK_:II.4 so it is highly likely that the *EPHB4* variant is causing the overlapping features in the two.

### Analysis of protein expression levels, kinase activity, and subcellular localization of EPHB4 mutated proteins expressed in LECs

Functional analysis of the two pathogenic missense variants originally reported to cause LRFH demonstrated normal EPHB4 protein expression levels but reduced protein activity.^2^ The analysis of 4 of the 20 missense variants reported to cause CM-AVM2 by Amyere et al. showed largely reduced protein expression levels with EPHB4 protein aggregated in intracellular inclusions.^6^ These findings led us to carry out a functional classification of the seven new variants for FH1-FH5, VA1–2, and PL1.

As a reference, the two previously published missense variants, p.R739Q and p.I782S, were included,^2^ and missense variant, p.D802G,^5^ was included as a representation of the CM-AVM2 phenotype. All three are located in the tyrosine kinase domain of EPHB4 in highly conserved residues and are predicted to be pathogenic by in silico analysis (Table 1, Supplementary Figures 4, 5). They are all novel, except p.D802G, which has been reported once in gnomAD, but is extremely rare (minor allele frequency [MAF] = 0.000004).

In addition, three variants located in the tyrosine kinase domain and not reported to be associated with disease were identified in gnomAD and included as controls. Two of the variants have been reported in homozygotes, they all had a low MAF (0.000024–0.006) and whilst we suspect them to be benign variants, in silico analysis predicts them to be pathogenic (Table 1, Supplementary Figures 4, 5A).

**Fig. 3:**
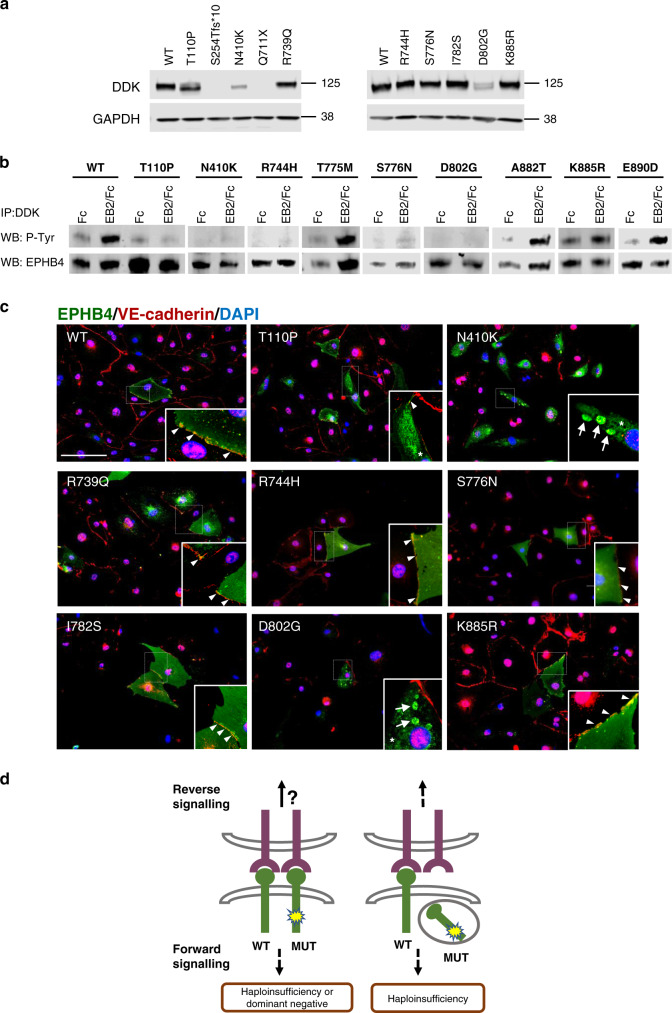
In vitro functional characterization of *EPHB4* VUS after transfection into lymphatic endothelial cells (LECs). (**a**) Western blot analysis of EPHB4 expression detected with anti-DDK antibody and GAPDH used as a loading control. The position of molecular mass markers (in kDa) is indicated to the right of the gel. (**b**) Effect of *EPHB4* variants on EPHB4 tyrosine phosphorylation in LECs after EphrinB2 stimulation with 1 μg/ml clustered Ephrin B2/Fc (EB2/Fc) or Fc alone. Receptor phosphorylation was analyzed by immunoprecipitation with anti-DDK antibody and western blotting using anti-p-tyrosine (upper panel) and EPHB4 (lower panel) antibodies. (**c**) Subcellular localization of EPHB4 variants. EPHB4 receptor was visualized with anti-DDK antibody (green), cell–cell contact with anti-VE-cadherin antibody (red) and nuclei with DAPI (blue). Membrane localization of the receptor is marked with arrowheads, cytoplasmic reticular localization with an asterisk, and intracellular aggregates with arrows. Images taken at 20× magnification with an EVOS™ M5000 imaging system. Scale bar 125 µm. For (**a**–**c**), one representative of ≥3 experiments is shown. (**d**) Model for potential EPHB4 molecular disease mechanisms. Left: Some mutant receptors were present in the membrane, lacking tyrosine activity. Whether this leads to EPHB4 haploinsufficiency or a dominant negative effect of the mutant EPHB4 was not tested in this study. Most variants fitting with this model were cases with a lymphatic-related fetal hydrops (LRFH) phenotype. Right: Other mutant receptors were sequestered into intracellular aggregates, leading to a loss-of-function disease mechanism. Most variants fitting with this model were cases with a CM-AVM2 phenotype. Bidirectional Eph-Ephrin signaling is complex with the existence of receptor–ligand oligomerization and bidirectional endocytosis of receptor–ligand complexes and we can only speculate on how forward or reverse signaling would be affected. MUT mutant EPHB4 receptor, WT wild-type EPHB4 receptor.

We generated mammalian expression vectors for the wild-type (WT) form of EPHB4 and for each of the 13 variants listed in Table 1 by site-directed mutagenesis. The constructs were transfected into LECs and the effects of the variants on EPHB4 protein expression levels were analyzed by western blot. Most of the variants tested resulted in expression levels comparable with the WT protein (Fig. 3a). However, the three variants, p.T110P, p.N410K, and p.D802G, showed reduced expression of protein, and what seems to be a shorter protein (compared with the predicted 108-kDa full-size protein). Variants p.Q711X and p.S254Tfs*10 could not be detected with the C-terminal specific antibody, indicating a loss of full-size protein expression. The in silico prediction from MutationTaster suggests the two variants lead to nonsense-mediated decay (NMD) due to the introduction of premature stop codons.

To investigate the possible impact of the variants on EPHB4 function, the tyrosine kinase activity of the receptor was analyzed by immunoprecipitation and western blot after EphrinB2 stimulation. We also included the three control variants in this analysis. Stimulation with preclustered EphrinB2 (EphrinB2-Fc) for 30 minutes increased the tyrosine phosphorylation level of the receptor after its binding in the WT form while tyrosine phosphorylation was reduced or not detected for most variants (Fig. 3b). The three control variants displayed tyrosine phosphorylation activity after EphrinB2 treatment; so did one VUS (p.K885R).

Immunofluorescence studies were performed to investigate the subcellular localization of the generated variants. Exogenous EPHB4 expression was detected with an anti-DDK antibody, while VE-cadherin expression was used as a marker of cell–cell membrane contact. Five of the variants investigated were detected in the cell membrane, confirmed by the localization in proximity to VE-cadherin, showing a similar expression localization pattern to the WT protein. On the other hand, the three variants, p.T110P, p.N410K, and p.D802G, showed absent or much reduced membrane localization and a distinct intracellular expression pattern. In some cells, increased reticular staining and/or intracellular aggregates that could be accumulations of misfolded proteins were detected when transfecting these three variants (Fig. 3c). A summary of our interpretation on the effect of the *EPHB4* variants regarding the expression levels, kinase activity, and subcellular localization obtained from the functional assays is shown in Supplementary Table 3.

## DISCUSSION

Since the initial reports of germline variants in *EPHB4 *associated with a disease phenotype,^2,4–9^ new cases with *EPHB4* VUS have come to our attention from several clinicians looking for additional evidence to confirm diagnosis. To verify the pathogenicity of all the new variants, we investigated protein expression levels, tyrosine kinase activity, and subcellular localization of the EPHB4 receptor. We showed that six of the seven novel variants under investigation resulted in a reduction or total absence of EPHB4 kinase activity, due to the presence of a dysfunctional receptor or total loss of protein expression. Thus, for most variants we were able to report a damaging effect on the protein function to the clinicians, suggesting that the VUS was pathogenic and causative of the disease. However, the p.K885R variant in family FH5 performed similarly to the WT protein in our functional assays. Based on our results, this variant appears benign and we cannot conclude it was the cause of the LRFH in this family; therefore, more extensive functional work must be done to confirm its pathogenicity.

We phenotypically describe eight new families with variable lymphatic and vascular phenotypes associated with novel *EPHB4* variants (summarized in Table 2) and provide a more detailed clinical report for the two families first published in 2016. Until now, our understanding has pointed toward the existence of two phenotypically differentiated autosomal dominantly inherited disease entities caused by germline variants in *EPHB4*: LRFH and CM-AVM2. Based on the evaluation of the new families, individuals in families VA1 and VA2 could be classified under the umbrella of CM-AVM2 as their presentation is similar to that given by Wooderchak-Donahue et al., who reported a mucocutaneous telangiectasia in a series of atypical HHT cases with *EPHB4* variants.^9^

**Table 2:** Clinical summary.

Overview of the phenotype of the eight new index cases and affected family members with an *EPHB4* VUS (FH1–FH5, VA1–VA2, and PL1) included in this study. Clinical details for the *EPHB4* index cases and affected family members previously reported by Martin-Almedina et al. (GLD_UK_ and GLD_NOR_)^2^ have been updated and included for comparison. Telangiectases are indicated in teal. Features originally described as associated with lymphatic-related fetal hydrops (LRFH) are indicated with a pale yellow. The lymphedema observed in PL1 is different on lymphoscintigraphy to that of individuals from the GLD_UK_ and GLD_NOR_ families, thus a darker shading has been used to indicate it. Refer to the Supplementary Information and Supplementary Table 1 for detailed clinical description of each case. “?” in the fetal hydrops column indicates that there were hydropic features at birth. “Normal” in the congenital heart defect column indicates that a normal echocardiogram was obtained. “(Yes)” in the persistent peripheral lymphedema column indicates that the individual is clinically normal, but abnormal lymphatic drainage was demonstrated on lymphoscintigraphy. Blank fields indicate the relevant procedure was not carried out or the information was not available.

*F* female, *FH* fetal hydrops, *GLD* generalized lymphatic dysplasia, *IUD* intrauterine death, *M* male, *ND* neonatal death, *PE* pleural effusions, *PL* primary lymphedema, *VA* vascular anomaly.

^a^Monozygotic twins.

^b^The type of *EPHB4* variant suggests this could be a capillary malformation–arteriovenous malformation 2 (CM-AVM2) case, but telangiectasia was not confirmed.

Five of the new cases were more difficult to interpret. They had fetal hydrops (FH1–FH3 and FH5) or pleural effusions (FH4), which were suspected to be lymphatic-related in FH1 and FH5 as they had chylous (“milky”) fluid drained postnatally. However, the lack of clinical information for the other cases does not allow us to conclude whether lymphatic dysfunction is the source of the fetal hydrops phenotype.

Of particular significance is the discovery of intrafamilial overlapping phenotypes with individuals presenting with features of both LRFH and CM-AVM2 (Table 2). An increasing number of allelic conditions have now been shown to have features of two different phenotypes in the same individual or family—the Janus (after the ancient Roman god with two faces) or dual phenotype.^23,24^ Furthermore, overlapping lymphatic and vascular features have previously been reported in patients with *RASA1*-associated CM-AVM type 1.^25^

Three individuals from the PL1 family presented with peripheral primary lymphedema but no history of fetal hydrops or ASD. The onset of lymphedema was around the age of 2 years and the lymphoscintigraphy was distinct from those of the LRFH families. PL1 also differs from the CM-AVM2 cases as there were no CM or AVMs. This is the first family to be described with just primary lymphedema and a likely pathogenic variant in *EPHB4*. Either early onset isolated peripheral primary lymphedema is a new separate entity (in addition to LRFH and CM-AVM2) in the increasing family of *EPHB4*-associated phenotypes, or, based on the proposed disease mechanism elucidated through our functional analysis, the CM-AVM2 phenotype spectrum needs expanding to include primary lymphedema.

Our results show different possible molecular disease mechanisms associated with EPHB4 pathogenesis and highlight the importance of further research into the functional and clinical consequences of the disruption of the EPHB4 forward and reverse signaling cascades. For some variants, we have shown that the EPHB4 mutant protein is expressed and presented on the membrane of LECs, but that tyrosine kinase activity is reduced, presumably altering the forward signaling (Fig. 3d, left). Reverse signaling might be unaffected although this was not investigated here. The reduced forward signaling could be due to an antimorphic disease mechanism with a dominant negative effect of the EPHB4 mutant on the WT protein activity. This dominant negative disease mechanism has been suggested for some *VEGFR3* variants associated with primary lymphedema.^26^ The variants in this category predominantly associated with LRFH with fetal hydrops, lymphovenous abnormalities, and ASD, some with very mild symptoms (e.g., FH3).

For other variants, we detected a loss of EPHB4 protein, probably due to either nonsense-mediated messenger RNA (mRNA) decay or protein degradation. Any EPHB4 mutant protein detected was of a shorter size and was trapped intracellularly, which could be a sign of the protein undergoing degradation. These EPHB4 mutants are most likely amorphic, acting as null alleles (Fig. 3d, right). Lower EPHB4 receptor levels will lead to an imbalance in the ratio of EphrinB2 ligand to EPHB4 receptor, which promotes cell dysfunction in normal microvascular endothelial cells.^27^ Variants in this category predominantly associated with CM-AVM2, but there were exceptions (e.g., the PL1 family). Bidirectional Eph-Ephrin signaling is complex and we can only speculate on how downstream forward or reverse signaling is affected by the *EPHB4* variants as this was not investigated in this study. The existence of receptor–ligand oligomerization and bidirectional endocytosis of receptor–ligand complexes add another level of intricacy, which supports the need for more research into the field to fully understand the contribution of both forward and reverse signaling to the arteriovenous and lymphovenous compartments.

Further studies are also needed to explain why the phenotype within families can be so variable. Several authors have suggested that a postzygotic de novo variant in the other allele (“second hit hypothesis”) would be required for the development of the focal lesions associated with the CM-AVM2 disease phenotype.^6–8^ This mechanism has been shown for other hereditary multifocal vascular malformations such as *RASA1*-associated CM-AVM1.^28–30^ However, variable expression of the disease phenotype could also be due to genetic modifiers or polygenic inheritance, therefore, it is necessary to understand whether one or two *EPHB4* null alleles will lead to disease.

In conclusion, several reports have shown that monoallelic *EPHB4* variants can cause a spectrum of arteriovenous and lymphovenous disorders, but our findings add more complexity to this. We introduce a possible new phenotype, dominant primary lymphedema, which showed no overlap with LRFH and CM-AVM2 as we currently understand them. However, with the observation of overlapping phenotypes between LRFH and CM-AVM2, the *EPHB4*-associated disease classifications might need a major revision. Regardless, a change of clinical practice is required as cases thought to be CM-AVM2 should undergo echocardiography, while cases thought to be LRFH should also undergo careful skin examination and consideration of brain and spine MRI to check for arteriovenous malformations. Furthermore, *EPHB4* variant pathogenicity was demonstrated in this study for six of the seven novel VUS investigated and highlights the usefulness of protein expression and subcellular localization studies to predict the pathogenesis of VUS. With NGS as a routine method for the diagnosis of inherited disorders, the detection rate for rare and novel variants is growing and inferring the pathogenicity of the identified variants can be challenging. Therefore, as suggested by the American College of Medical Genetics and Genomics/Association for Molecular Pathology (ACMG/AMP) guidelines,^31^ a comparable approach is essential for other genes/diseases to maximize diagnostic rates. Our study also highlights different *EPHB4*-related disease mechanisms; however, further research is necessary to expand our knowledge on how they operate at a cellular and molecular level to fully understand the intricate EPHB4 clinical enigma.

## Supplementary information

Supplementary Table 1

Supplementary Information

## Data Availability

The most recent version of the scripts used for the described analysis can be found online (Preprocessing/Alignment: https://github.com/sgul-genetics-centre-bioinformatics/Next-Generation-Sequencing-Pipelines; Unpaired Somatic Calling: https://github.com/digrigor/Unpaired_somatic_variant_calling. Archived version of this software can be found here (https://figshare.com/s/ecf5dcb85b7420be4938).
