# Correction: Janus-faced *EPHB4*-associated disorders: novel pathogenic variants and unreported intrafamilial overlapping phenotypes

**DOI:** 10.1038/s41436-021-01202-0

**Published:** 2021-05-26

**Authors:** Silvia Martin-Almedina, Kazim Ogmen, Ege Sackey, Dionysios Grigoriadis, Christina Karapouliou, Noeline Nadarajah, Cathrine Ebbing, Jenny Lord, Rhiannon Mellis, Fanny Kortuem, Mary Beth Dinulos, Cassandra Polun, Sherri Bale, Giles Atton, Alexandra Robinson, Hallvard Reigstad, Gunnar Houge, Axel von der Wense, Wolf-Henning Becker, Steve Jeffery, Peter S. Mortimer, Kristiana Gordon, Katherine S. Josephs, Sarah Robart, Mark D. Kilby, Stephanie Vallee, Jerome L. Gorski, Maja Hempel, Siren Berland, Sahar Mansour, Pia Ostergaard

**Affiliations:** 1 Molecular and Clinical Sciences Institute, St George’s University of London, London, UK; 2 Department of Obstetrics and Gynecology, Haukeland University Hospital, Bergen, Norway; 3 Wellcome Sanger Institute, Hinxton, UK; 4 North Thames Genomic Laboratory Hub, Great Ormond Street Hospital for Children NHS Foundation Trust, London, UK; 5 Genetics and Genomic Medicine, UCL Great Ormond Street Institute of Child Health, London, UK; 6 Institute of Human Genetics, University Medical Center Hamburg Eppendorf, Hamburg, Germany; 7 Departments of Pediatrics – Section of Genetics and Child Development, Dartmouth-Hitchcock Medical Center, Lebanon, NH USA; 8 Geisel School of Medicine at Dartmouth College, Hanover, NH USA; 9 Department of Child Health, University of Missouri School of Medicine, Columbia, MO USA; 10 GeneDx, 207 Perry Parkway, Gaithersburg, MD USA; 11 University Hospitals Bristol NHS Foundation Trust, Bristol, United Kingdom; 12 Neonatal intensive care unit, Children’s Department, Haukeland University Hospital, Bergen, Norway; 13 Department of Medical Genetics, Haukeland University Hospital, Bergen, Norway; 14 Department of Neonatology and Paediatric Intensive Care, Altona Children’s Hospital, Hamburg, Germany; 15 Elbe Center for Prenatal Medicine, Hamburg, Germany; 16 Dermatology & Lymphovascular Medicine, St George’s Universities NHS Foundation Trust, London, UK; 17 South West Thames Regional Genetics Service, St George’s NHS Foundation Trust, London, UK; 18 The Institute of Metabolism & Systems Research, College of Medical & Dental Sciences, University of Birmingham, Birmingham, UK; 19 West Midlands Fetal Medicine Centre, Birmingham Women’s & Children’s Foundation Trust, Birmingham, UK

Correction to: *Genetics in Medicine* 2021; 10.1038/s41436-021-01136-7; published online 16 April 2021

Due to a processing error, the ESM was incomplete. The complete ESM is given on 10.1038/s41436-021-01136-7.

Unfortunately an error occurred in Fig. 1a. The corrected Fig. 1 is given below:

**Fig. 1:**
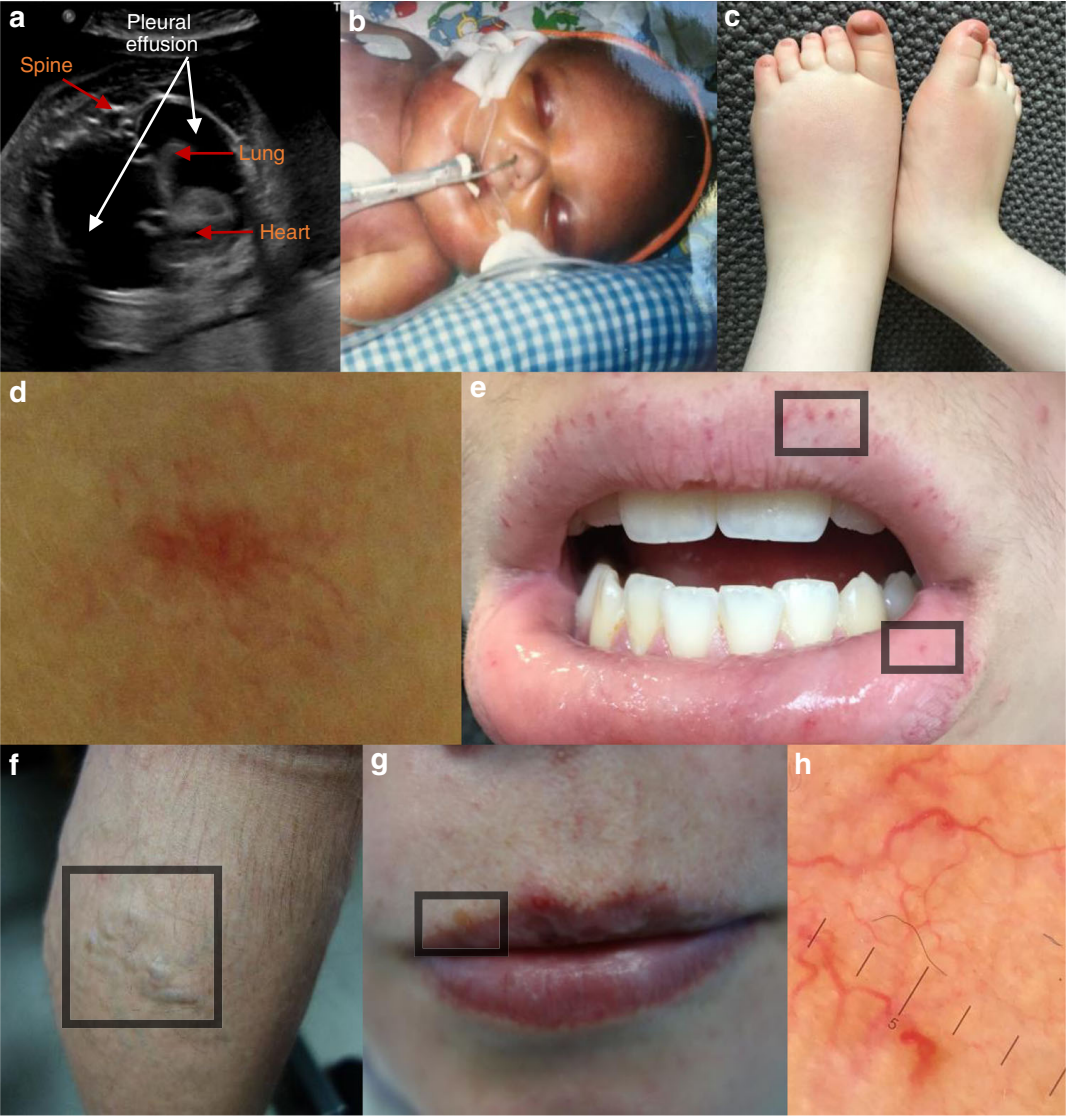
Clinical findings in individuals with *EPHB4* variants. (**a**) Antenatal ultrasound scan (transverse plane) demonstrating bilateral pleural effusions at gestational week 30 + 4 in FH2:II.1. (**b**) Baby in the neonatal period with fetal hydrops (GLDUK:II.6). (**c**) Persistent peripheral lymphedema in the feet of FH5:II.2 at age 4 years. (**d**) Capillary malformation in the midline of the neck in VA1:II.2. (**e**) Multiple telangiectasia along the vermilion border of the upper lip and the mucous membrane of the lower lip in VA2:II.1 (inside boxed areas). (**f)** Early onset and extensive lower limb varicose veins in GLDUK:I.2. (**g**) GLDUK:II.4 with multiple telangiectasia with a propensity for the vermilion border of the lips (inside boxed area). (**h**) Dermatoscopic image of telangiectasia on the left cheek confirming the presence of dilated linear and branching capillary vessels in GLDUK:II.4.

The original article has been corrected.

