# Supplementary material for: Janus-faced EPHB4-associated disorders: novel pathogenic variants and unreported intrafamilial overlapping phenotypes
Source: Genet Med. 2021 Apr 16;23(7):1315–24. doi: 10.1038/s41436-021-01136-7 (PMC8257501; doi:10.1038/s41436-021-01136-7)
Supplement: Supplementary file 2 — Supplementary Information [file 41436_2021_1136_MOESM2_ESM.pdf]

Supplementary Information

Janus-faced *EPHB4*-associated disorders – novel pathogenic variants and unreported intrafamilial overlapping phenotypes

Silvia Martin-Almedina PhD[1], Kazim Ogmen PhD[1]*, Ege Sackey PhD[1]*, Dionysios Grigoriadis MS[1], Christina Karapouliou PhD[1], Noeline Nadarajah MS[1], Cathrine Ebbing MD[2], Jenny Lord PhD[3], Rhiannon Mellis MD[4,5], Fanny Kortuem MD[6], Mary Beth Dinulos MD[7,8], Cassandra Polun MS, LCGC[9], Sherri Bale PhD[10], Giles Atton MD[1], Alexandra Robinson MD[1,11], Hallvard Reigstad MD[12], Gunnar Houge MD[13], Axel von der Wense MD[14], Wolf-Henning Becker MD[15], Steve Jeffery PhD[1], Peter Mortimer MD[1,16], Kristiana Gordon MD[1,16], Kat Josephs MD[1,17], Sarah Robart MD[4], Mark Kilby MD[18,19], Stephanie Vallee MD[7], Jerome Gorski MD[9], Maja Hempel MD[6], Siren Berland MD[13], Sahar Mansour MD[1,17‡], Pia Ostergaard PhD[1‡].

Clinical report

The clinical details for the ten index cases with *EPHB4* variants and their affected family members are given below. Pedigrees are available in Supplementary Figures 1 – 3 and clinical details are summarised in Supplementary Table 1.

Lymphatic-Related Fetal Hydrops (LRFH) cases

GLD_{UK} with EPHB4: c.[2216G>A];[=]; p.[Arg739Gln];[=]

This family (GLD_{UK}) was ascertained from the specialist primary lymphedema clinic at St George's Hospital, London, UK. Seven members of the family were formally assessed. This family has previously been reported by Martin-Almedina *et al.* (1).

GLD_{UK}:I.2 was born prematurely at 36 weeks, weighing 2.24kg (9th centile) and was not edematous. The neonatal period was uneventful. In early childhood, he was diagnosed with a large atrial septal defect (ASD) which was surgically corrected aged 6 years. Between the ages of 11-13 years he had

several admissions to hospital with ‘pleurisy’. At 15 years old he rapidly developed persistent swelling of his left leg, followed by right leg swelling aged 18 years. From age 24-42 years he experienced recurrent lower limb cellulitis requiring multiple hospital admissions. At last follow-up (age 55) he has bilateral lower limb lymphedema, more pronounced on the right, with extensive varicose veins (Figure 1F). He has deep-set nails, deep interphalangeal creases, and a positive stemmer-sign bilaterally (an inability to pinch the skin on the dorsum of the second toe, indicative of lymphedema). Compression stockings are the mainstay of his treatment. Lower limb lymphoscintigraphy results are consistent with bilateral lymphedema, with tracer retention at the site of injection and markedly reduced uptake of tracer particularly in the left leg where there is no visible uptake of tracer in the left inguinal lymph nodes (Figure 2G) (1). There is evidence of superficial rerouting/reflux of lymph through collateral channels in the right lower leg and deep rerouting via the right popliteal lymph nodes. Tracts in the right leg are also tortuous. Further genetic analysis of GLD_{UK}:I.2 suggests that he is possibly mosaic as the exome sequencing data indicates 37% of mutant reads and 63% wild-type (WT), confirmed by a somatic variant calling analysis (Supplementary Figure 6).

GLD_{UK}:II.2 presented antenatally with fetal hydrops, including subcutaneous edema, bilateral pleural effusions and ascites. Antenatal karyotyping was normal (46,XX). She was born prematurely at 34 weeks, initially weighing 3.4kg (>99.6th centile) before losing weight to 1.4kg (<3rd centile) as her edema resolved. She had a complicated neonatal period, requiring ventilation for 14 weeks and dialysis for renal failure. She had persistent pleural effusions, ascites and neck swelling but no peripheral lymphedema. She was diagnosed with a congenital diaphragmatic hernia and an ASD, which were both surgically corrected. Additional abnormalities include a structurally normal ectopic kidney in the left iliac fossa, bilateral strabismus and splenomegaly. On examination at age 28 she had mild orbital hypotelorism, posteriorly rotated ears, hypermobile joints and nasal speech. She has mild learning difficulties, most likely secondary to hypoxia during the neonatal period. Clinically she lacked typical peripheral lymphedema but had ‘full’ calves. Despite this, lower limb

lymphoscintigraphy demonstrated bilateral, symmetrical impaired lymphatic drainage with retention of tracer at the injection site after 2 hours (Figure 2F). The main lymphatic tracts are tortuous. She has quite extensive varicose veins and at the most recent visit (age 35y) no telangiectases were detected.

GLD_{UK}:II.4 was born at 38 weeks following an uneventful pregnancy with no evidence of fetal hydrops. She had no medical problems prior to an echocardiogram at age 23, which detected a small ASD. She has no peripheral edema, however, lymphoscintigraphy showed some rerouting of lymph transport in the calves only and tortuous lower limb tracts which could indicate venous hypertension (Figure 2E) (1). There was no evidence of impaired lymphatic drainage as quantification of uptake of tracer in the inguinal lymph nodes is within the normal range. However, the 15-min scan (not shown) showed abnormal early drainage of tracer to both knees and therefore, the 2-hour quantification figures are likely to be falsely elevated due to venous hypertension. She does have varicose veins and a venous duplex scan confirmed this in the right leg.

She was re-examined recently (at age 29y) because her son (GLD_{UK}:III.2) was suffering from recurrent nose bleeds. She was found to have diffuse, multiple telangiectasia over her cheeks and lips, and a few on the upper arms and hands but not involving the mucus membrane (Figure 1G, H). There were no other capillary malformations. An MRI of the brain and spine was normal and showed no vascular malformations. No pathogenic variants were identified in the known hereditary haemorrhagic telangiectasia (HHT) genes: *ACVRL1, ENG, GDF2, SMAD4* or in *RASA1*.

GLDUK:II.6 had a slightly increased nuchal translucency (NT) (3.0mm, normal <2.5mm) at 13 weeks gestation. Antenatal echocardiography and karyotype (46,XY) were normal. A right pleural effusion was detected at 20 weeks, progressing to large, bilateral pleural effusions and a small pericardial effusion by 29 weeks. His bilateral pleural effusions required drainage with pleuro-amniotic shunts (right at 23 weeks, left at 29 weeks). He was born prematurely by emergency caesarean section at 34 weeks due to preeclampsia. It is likely his mother had mirror syndrome, a rare disorder where

there is fetal and placental hydrops with maternal edema. At birth he was edematous with a weight of 3.7kg (>99.6th centile) and required immediate intubation (Figure 1B). He was dysmorphic with low set ears, hypertelorism and a depressed nasal bridge, possibly due to edema. His health deteriorated over the first three weeks of life with refractory hypotension, renal failure, and metabolic acidosis. He developed severe generalized edema with marked hypoalbuminaemia. He died aged 21 days.

GLD$_{UK}$:II.7 was found to have an increased NT (3.8mm) at 12+4 weeks gestation which persisted and increased. Antenatal echocardiography and karyotype (46,XX) were normal. Severe fetal hydrops was diagnosed at 20 weeks with cutaneous edema, massive bilateral pleural effusions and ascites. Pleuro-amniotic shunts were inserted with some initial success in reducing edema. However, the mother developed edema at 25 weeks gestation likely due to mirror syndrome. Despite the initial improvement, the baby died *in utero* at 26+4 weeks gestation.

GLD$_{UK}$:III.1 had a borderline high NT of 2.8mm at 12 weeks. At 20 weeks, there was persistent nuchal edema with a moderate pericardial effusion which resolved by 22 weeks. However, by 30 weeks gestation, there was a right-sided pleural effusion which required drainage and a small left sided pleural effusion found at 31 weeks. There was no ascites or skin edema. Antenatal karyotyping was normal (46,XY). He was born prematurely at 32 weeks gestation with a normal birth weight of 2.02kg. He had persistent pleural effusions for which he required artificial ventilation and bilateral drainage but no peripheral swelling. The pleural fluid was chylous, so he was treated with a low-fat diet rich in medium-chain triglycerides and octreotide until resolution of the pleural effusions at 8 months. Echocardiography identified an ASD which spontaneously closed later in childhood. On examination at 10 months of age he had no dysmorphic features and a weight and head circumference on the 2nd to 9th centile. He is cognitively normal with no evidence of peripheral or systemic lymphatic disease.

GLD_{UK}:III.2 had no antenatal edema and was born by vaginal delivery at term following an uneventful pregnancy. His birthweight was 4.37kg but he lost over 10% of this to 3.6kg in the first few days. Echocardiogram identified two ASDs (9 mm and 3 mm) which were surgically closed at 3 years of age. Prior to this he had recurrent chest infections and marked sternal recession. At age 4 years he was not dysmorphic but had a mild pectus excavatum and a social communication disorder with speech and language delay. There was no peripheral edema. At the age of 9 years he developed recurrent epistaxis. Examination at that stage identified telangiectasia on his lower lip, left upper arm and right nipple confirmed by dermoscopy. No brain or spine AVMs were detected on MRI.

GLD_{NOR} with EPHB4: c.[2345T>G];[=]; p.[Ile782Ser];[=]

This family (GLD_{NOR}) was previously reported by Martin-Almedina *et al.* and has been followed at Haukeland University Hospital, Bergen, Norway (1). Four members of the family were formally assessed. Monozygotic twins (GLD_{NOR}:II.2 and GLD_{NOR}:II.3) were born spontaneously at 34 weeks gestation. They were confirmed to be monozygotic by zygosity testing of DNA from peripheral blood. They were diamniotic and dichorionic, with normal placentae with a combined weight of 1500g.

GLD_{NOR}:II.2, the first twin, had Apgar scores of 6 and 8 after 1 and 5 minutes (normal) and weighed 1.76kg (9th-25th centile). She had mild respiratory distress requiring supplemental oxygen for 2 days, though an initial chest radiograph was normal. Edema was present in the lower extremities and the right arm for a few days only, with normal albumin level at day 2 (30 g/L), and mild hyperbilirubinemia (highest level 220 µmol/l) treated with UV-light. Her haemoglobin concentration was slightly low at birth (172 g/L, normal 193 ± 2.2 g/L) but fell to 89 g/L at 2 days of age. This remained low at her 11-day follow-up (73 g/L) and she was given one blood transfusion. Following this she had an uneventful childhood.

Her obstetric history was remarkable with a total of 6 pregnancies, including 3 first trimester miscarriages and a termination of pregnancy at 20 weeks of gestation for trisomy 18 (karyotype 47,XX+18). In the fifth pregnancy she gave birth to a son (GLD_{NOR}:III.5) by elective caesarean section

at 30+4 weeks who died at 36 hours (see below). One year later she gave birth to a healthy son after an uneventful pregnancy and birth.

At the most recent clinical examination of GLD_{NOR}:II.2 (age 37) her height was 161.5 cm (40th centile), her head circumference was 53.5 cm (25th centile) and there were no dysmorphic features. Clinically there was no peripheral swelling, but lower limb lymphoscintigraphy showed multiple tortuous lymphatic tracts in the lower limbs and superficial rerouting of tracer is apparent in both calves (Figure 2C). There was delayed uptake of tracer in the left ilioinguinal nodes and borderline on the right. She has bilateral varicose veins in the lower extremities, present since age 23. At age 37 the severity of the varicose veins were mild/moderate and graded as C3 on left leg (edema secondary to varicose veins) and C2 on the right (asymptomatic), with insufficiency of the great saphenous vein bilaterally (highest diameter 13mm left and 7 mm right, normal average 5.0 ± 2.4 mm). At age 37 she had normal echocardiography (no ASD), normal renal function and she is otherwise healthy. She had recurrent nose bleeds in childhood, but never problematic. However, she is not clinically assessed for telangiectases so the nose bleeds could be unrelated.

GLD_{NOR}:II.3 was the second twin. She was born by vaginal delivery at 34 weeks with breech presentation. She had low Apgar scores of 2 and 2 after 1 and 5 minutes and weighed 2.0kg (25th-50th centile). At clinical examination 1.5 hours after birth, she had pitting edema, peripheral cyanosis, no movements except infrequent spontaneous respiration, low heart rate (100 beats/minute, normal 120-160 beats/minute) and she needed continuous ventilation, for a total of 3 weeks. Her chest radiograph showed extensive pulmonary edema with pleural effusions. She also had increasing edema in the first 4 days, most pronounced in the trunk, abdomen and face. Multiple thoracocentesis procedures were required; blood stained straw-coloured pleural fluid was drained bilaterally on day 1. Following this, full enteral nutrition was given. Repeat thoracentesis on day 28 produced milky fluid containing high triglycerides (225 mg/dL) and low cholesterol (1.4 mmol/L), confirming that it was a chylous effusion, which is diagnosed at triglycerides >110 mg/dL and

cholesterol <5.18 mmol/L. Dietary treatment was started from day 28 using an enteral low-fat diet with medium chain triglycerides, guided by Gershanik *et al.* (2). She was anaemic at birth (152 g/L, normal 193 ± 2.2 g/L) and was transfused 6 units of red blood cells in the first month and a further one at 65 days of age. The serum albumin level at birth was low (17g/L; normal range 35-50g/L) and she had one infusion of albumin 200mg/ml. She was discharged at 2 months of age, with full resolution of the pleural effusions at 3 months of age, allowing her medium chain triglyceride diet to cease.

Despite a number of hospital readmissions with respiratory infections in the first year of life, she achieved normal developmental milestones. She has uterus didelphys with a vaginal septum. She had two first trimester miscarriages. She then gave birth to a son with severe chylothoraces who survived (GLD_{NOR}:III.9, see below) and a second son who was healthy.

At the most recent clinical examination of GLD_{NOR}:II.3 at age 37 her height was 159 cm (25th centile) and head circumference 53.5 cm (25th centile). She has no dysmorphic features. Clinically she has no lymphedema, but lower limb lymphoscintigraphy showed bilateral tortuous tracts (Figure 2D). Superficial rerouting of tracer is present around the ankles and calves. The uptake of tracer in the ilioinguinal nodes after 2 hours was within the normal range. She has had varicose veins in the lower extremities since her late teens, for which she has had numerous operations. A duplex ultrasound (US) scan at age 29, before pregnancy, showed deep venous insufficiency of the great saphenous vein bilaterally and small saphenous vein on the left. Echocardiography at age 37 was normal except for an insignificant patent foramen ovale (PFO). She had recurrent heavy nose bleeds in her childhood (until the age of 9), which happened more often and with a prolonged duration compared to her sister and other family members, however, she is not clinically assessed for telangiectases so the nose bleeds could be unrelated. Her renal function is normal, and she is otherwise healthy.

GLD_{NOR}:III.9: There were no concerns in the first trimester of GLD_{NOR}:II.3's third pregnancy, with a normal NT measurement (1.8mm) at 12 weeks gestation followed by a normal second trimester US

scan. Due to her uterus didelphys, she had a follow-up US scan at 28 weeks for evaluation of fetal growth and premature birth risk assessment. Fetal hydrops was diagnosed with bilateral pleural effusions, subcutaneous edema on the upper part of the body, polyhydramnios and a thick placenta was noted. The abdominal circumference was large (99th centile) compared with normal head and femur size. This suggested possible hepatomegaly. There was an anomalous course and length of the connection between the intra-hepatic umbilical vein/portal system and the inferior vena cava, suggesting a portosystemic shunt. Doppler analysis found flow velocity lower than normal in the ductus venosus, indicating a low portocaval pressure gradient, and pulsatility higher than normal in the ductus venosus, a likely sign of developing cardiac failure.

Thoracocentesis was performed four times between 28 and 30 weeks gestation. In the first of these, the pleural fluid showed highly elevated leucocytes, 97% of which were mononuclear cells, consistent with lymphedema. A small amount of ascites around the liver was noted, but responded intermittently to drainage. Caesarean section was performed at 30+4 weeks gestation, due to premature rupture of the membranes and increasing signs of fetal cardiac failure; reversed end-diastolic flow in the umbilical arteries, also indicative of placental disease.

GLD_{NOR}:III.9 had low Apgar scores (2, 7 and 8 at 1, 5 and 10 mins), his birth weight was 1.58 kg (50th - 75th centile) and his length 40 cm (25th to 50th centile). He was intubated at birth and treated with high frequency ventilation. He had bilateral chest drains for four weeks. The pleural fluid on day 1 contained high leucocyte levels with 98% mononuclear cells, consistent with lymphatic fluid. He had some subcutaneous edema at birth, mostly around the neck, face and scrotum which did initially improve. However, after 2 weeks he had increasing respiratory distress. He developed renal failure; with a maximum creatinine of 225 µmol/L at day 14. He had considerable volume overload, which necessitated peritoneal dialysis for eight days. During this critical period, he developed massive edema. He was on an oscillator for three weeks, followed by conventional ventilation for four weeks, then nasal continuous positive airway pressure until 2 months of age. He was given total parenteral nutrition for three weeks, then a low-fat diet with medium chain triglycerides and ultimately

ordinary formula from 6 weeks of age. Serum albumin was low at birth (14 g/L, normal 35-50 g/L) which was replaced intravenously as substitution for lymphatic loss through pleural drainage. Albumin was low in the first 2.5 months. Haemoglobin levels were low at birth (169 g/dL, normal 193 ± 2.2 g/L), falling to 120 g/L at day 7, prompting blood transfusions at days 2 and 7. Renal ultrasound revealed small kidneys with hyperechogenic cortex and pyramids. He also had surgery for bilateral inguinal hernias. Echocardiography on day 1 showed a small ASD and PFO that both spontaneously closed by follow-up at day 10.

GLD_{NOR}:III.9 had a normal karyotype. At the age of five years he had chronic kidney disease stage 3 with a glomerular filtration rate of 58.7 ml/min/1.73m^2, approximately 50% of the expected value. He had no learning difficulties. At age 7, he has a moderate sensorineural hearing loss (no pathogenic variant was identified in *GJB2* [connexin 26] or copy number alterations in *GJB6/2* and he was negative for a NGS panel for hearing loss). His kidney disease could be caused by acute tubular necrosis and hypotension in infancy, however, use of gentamicin and vancomycin, are also associated with both nephrotoxicity and ototoxicity. His development is only slightly delayed, notably his language, secondary to hearing loss, for which he uses occasional hand signs. He is otherwise normal. His height was 116.6 cm (3rd centile). He has no neck webbing or dysmorphic features, no edema and no obvious vascular anomalies. Similar to his mother GLD_{NOR}:II.3, he suffered from heavy nose bleeds, but no telangiectasia have been seen in the nasal mucus membrane, so could be unrelated.

GLD_{NOR}:III.5 is the fifth pregnancy of GLD_{NOR}:II.2. GLD_{NOR}:III.5 was born 2 weeks after his cousin, GLD_{NOR}:III.9. Due to the finding of fetal hydrops in GLD_{NOR}:III.9, GLD_{NOR}:II.2 was offered an US scan at 28 weeks gestation. This revealed an almost identical result of severe hydrops with bilateral pleural effusions, subcutaneous edema of the upper body and a thickened placenta. In contrast to her sister, GLD_{NOR}:II.2 had oligohydramnios. Like his cousin, this fetus had a large abdominal circumference, the ductus venosus had an anomalous course towards the central veins, and blood flow velocity pattern was similar to the cousin's, i.e. there was a low flow velocity and a high

pulsatility index in the ductus venosus. Also, a compensatory high hepatic artery contribution to liver perfusion was found, showing high blood velocity and low pulsatility index. Flow velocity in the umbilical vein at the abdominal wall was high and pulsatile, suggesting an umbilical vein constriction and heart failure. The situation was considered more severe for this fetus, which developed intrauterine growth restriction during the last weeks.

Following maternal treatment with bethametasone for fetal lung maturation, thoracocentesis was performed three times. The baby was delivered by caesarean section at 30+4 weeks gestation. He had an Apgar score of 7, 7 and 7 at 1, 5 and 10 minutes with a birth weight of 1.8kg (75th to 91st centile) and length was 37 cm (2nd to 9th centile). He was ventilated from birth and had bilateral chest drains. At birth, haemoglobin concentration was low at 158 g/L (normal 193 ± 2.2 g/L) and low albumin 15 g/L (normal 35-50 g/L). Pleural fluid (possibly taken prior to parenteral nutrition) contained leucocytes 1.6 mg/dL (71% mononuclear), cholesterol 0.4 mmol/L, triglycerides 0.48 mmol/L and protein 12 g/L consistent with a transudative effusion and ruled out a chylothorax, (diagnosed at triglycerides >1.24 mmol/L and cholesterol <5.18 mmol/L and mononuclear leucocytes >90%). Echocardiography showed a high perimembranous ventricular septal defect (VSD), a small ASD and PFO. Despite maximal respiratory support, the edema increased, and he died from severe respiratory distress and renal failure at 36 hours. Post mortem autopsy for GLD_{NOR}:III.5 was not performed, but DNA available for cytogenetic testing displayed a normal karyotype.

Non-Immune Fetal Hydrops cases of unknown etiology

FH1 with EPHB4: c.[2231G>A];[=]; p.[Arg744His];[=]

FH1:II.2 was ascertained from the neonatal intensive care unit of Women and Children's Hospital at the University of Missouri Medical System, USA. FH1:II.2 was the second child of non-consanguineous, Caucasian parents. There was no family history of note. The pregnancy was

complicated by pleural and pericardial effusions, first noted at 29 weeks gestation by prenatal US. Thoracentesis stents were placed prenatally at 29 weeks gestation.

He was born by normal vaginal delivery at 31 weeks gestation following premature rupture of membranes with presumed placental abruption. Apgar scores were 1, 5, and 6 at 1, 5, and 10 minutes, respectively. Following the delivery, the baby had left-sided thoracentesis performed, which yielded 60 mL of amber-coloured fluid.

At birth, his head circumference was 32 cm (>97th centile), length 48 cm (> 97th centile) and weight was 2.74kg (> 97th centile). These growth parameters would be more consistent with an infant of approximately 37 weeks gestation, not 31 weeks, and likely result from edema.

At birth, FH1:II.2 was markedly edematous with pitting edema on the scalp and trunk. He had bilateral epicanthal folds, an edematous nose with a shallow nasal bridge and edematous ears with over-folded helices. The abdomen was markedly protuberant with scrotal swelling. FH1:II.2 required maximal ventilatory support but developed increasingly widespread, generalized swelling. Analysis of fluid from thoracocentesis was consistent with chylothorax. Over the following weeks, he had thoracocenteses at least 4 times, with 3 chest drains placed and a Broviac subcutaneous port. A chest CT scan showed large, bilateral pleural effusions, right greater than left, that appeared to be loculated. FH1:II.2 presented with severe respiratory distress syndrome, complicated by his pleural effusions. Echocardiogram showed a PFO with increased right ventricular pressures. There was also a small pericardial effusion. Renal evaluation showed normal kidneys with normal renal function. As a complication of his neonatal problems, he developed a grade 4 intraventricular haemorrhage with myoclonic seizures. This manifested as back arching with apnoeic and bradycardic episodes. Electroencephalogram showed decreased cerebral activity with epileptiform foci in the right central and temporal regions with observed myoclonic jerks. FH1:II.2 failed extubation on several occasions and his medical course continued to deteriorate. He died at 36 days. Chromosomal microarray showed a normal male chromosome constitution without significant copy number variants.

FH2 with EPHB4: c.[2231G>A];[=]; p.[Arg744His];[=]

FH2:II.1 was ascertained from the University Medical Center Eppendorf, Hamburg, Germany. FH2:II.1 was conceived naturally and is the first child of healthy, unrelated Caucasian parents. There was no significant family history. Nuchal translucency (NT) was normal (2.0 mm) at 13 weeks gestation. During the routine 19-week fetal anomaly scan, a right pleural effusion was diagnosed. One week later this had almost resolved spontaneously. Extensive genetic testing gave normal results for karyotyping, CGH array and a RASopathy gene panel including *PTPN11, BRAF, KRAS, NRAS, SHOC2, SOS1, RAF1, RIT1, RRAS, CBL.* At 28 weeks gestation, a moderate right-sided pleural effusion and polyhydramnios were observed. At 30 weeks, generalized hydrops fetalis with a pronounced right-sided pleural effusion, ascites and subcutaneous edema had developed in addition to polyhydramnios (Figure 1A). Doppler signals of different blood vessels were reduced and partially reversed, indicating an impaired blood flow in the fetus.

FH2:II.1 was born prematurely at 30 weeks gestation by emergency caesarean section due to fetal bradycardia following placement of a pleuro-amniotic shunt. Her birth weight was 2.4 kg (>99th centile), length 45 cm (90th centile) and head circumference 32.5 cm (98th centile). Apgar scores were 4, 7 and 7 at 1, 5 and 10 minutes after birth, respectively. She presented with generalized subcutaneous edema, severe bilateral pleural effusions and ascites, requiring artificial ventilation and insertion of bilateral chest drains. Despite treatment, the pleural effusions, ascites and subcutaneous edema persisted, and she developed a pericardial effusion. A patent ductus arteriosus (PDA) was surgically closed at day 10. Unilateral pleurodesis was performed in her 6th week of life. Lung biopsy showed dysplastic alveoli with a rarefication of the alveolo-septal capillaries (a reduced number of capillaries in the membrane surrounding the alveolae). FH2:II.1 died at the age of 9 weeks.

FH3 with EPHB4: c.[2327G>A];[=]; p.[Ser776Asn];[=]. PAGE Study ID PP1474

FH3:II.7 was born to a Caucasian 34-year old mother (gravida 7, para 5, miscarriage x1). NT was normal (1.3mm) at the 12-week scan. At 26 weeks gestation, pleural effusions, a moderate right and

a small left, were diagnosed with slight mediastinal shift. PCR and array CGH results from amniocentesis at 27+0 weeks gestation were normal. Fetal echocardiogram at 29+0 weeks was normal. The pleural effusions gradually improved until 33 weeks gestation, at which point a small residual right pleural effusion remained and the patient was discharged back to her local hospital to continue the pregnancy. The female infant was born at term by normal vaginal delivery (birth weight 3340g) with no apparent abnormality reported but was, unfortunately, lost to further follow up.

*FH4 with EPHB4: c.[760_761insC];[=]; p.[Ser254Thrfs*10];[=]. PAGE study ID PP2904*

This male fetus, FH4:II.1, presented with an increased NT of 9.5mm (normal <2.5mm) on the 12-week scan. At 20 weeks non-immune fetal hydrops and an ASD were noted. Array CGH results from chorionic villus sampling were normal. The *EPHB4* variant was not detected in either parent. This is unfortunately the limit of the prenatal information and no outcome information is available possibly due to IUD.

FH5 with EPHB4: c.[2654A>G];[=]; p.[Lys885Arg];[=]

FH5:II.2 presented with non-immune fetal hydrops with ascites and subcutaneous edema noted on a 30-week prenatal US. He was hydropic at birth (30-week gestation) and fluid drained was chylous. He was thought to have a scalp lesion that resembled cutis aplasia congenita. He had a large PDA that has persisted (possibly an ASD). The hydrops resolved in infancy. FH5:II.2 is slightly dysmorphic with widened inner canthal distance, epicanthal folds, periorbital fullness. Peripheral swelling (lymphedema) of the lower extremities was noted at 7 months. The patient continues to have lymphedema of feet and mild periorbital edema at 4 years (Figure 1C). Hypotonia, hyperconvex nails and developmental delay was also noted as well as petechiae.

The *EPHB4* variant was maternally inherited but the mother (FH5:I.2) is asymptomatic. There was no history of hydrops, edema or lymphatic symptoms as a neonate, she does appear to have very mild varicose veins of her legs. No abnormalities were detected on echocardiogram.

The proband’s older sister (FH5:II.1) had “swelling” as an infant which resolved by 2 weeks of age. NT on a 12-week scan was normal. 3rd trimester ultrasounds showed that her abdominal circumference measurements were all >97th percentile. She was 38-week gestation and had marked periorbital edema at birth. Normal echocardiogram at 7 years.

Vascular Anomaly cases

VA1 with EPHB4: c.[2131C>T];[=] p.[Gln711Ter];[=]

VA1:II.2 is a 15-year-old girl who presented with telangiectases of the face, lips, and hands since the age of 3 years. In addition to the telangiectasia, she has capillary malformation on her neck, upper chest and back (Figure 1D). She suffers from recurrent nose bleeds up to five times per year and bruises easily. She has heavy, irregular periods. No pathogenic variant or copy number variants were identified in the known HHT genes (*ACVRL1, ENG, GDF2* or *SMAD4*) or *RASA1*.

Her mother (VA1:I.2) has telangiectases on her face and hands and her sister (VA1:II.1) has them on her face and right eye and a capillary malformation on her left upper arm. Both appear to be more mildly affected than the proband. They both have heavy irregular periods but no history of nose or gastrointestinal bleeding. Her mother (VA1:I.2) was treated for thyroid cancer, but no details are available. Both carry the same variant in *EPHB4* as the proband. An MRI of the brain and spine was normal and showed no vascular malformations any of the three.

VA2 with EPHB4: c.[328A>C];[=]; p.[Thr110Pro];[=]

VA2:II.1 presented with telangiectasia of the hands, lips and the mucus membrane of his lower lip from approximately the age of 14 years (Figure 1E). He had suffered from recurrent nose bleeds when younger (approximately one per month), but these had stopped at the age of 12 years. He was otherwise well with no gastrointestinal bleeding and no shortness of breath. No pathogenic variants or copy number variants were identified in the known HHT genes (*ACVRL1, ENG, GDF2* or *SMAD4*) or *RASA1*.

His father, VA2:I.1, was said to have had telangiectasia when younger but they were not visible on examination. He was otherwise fit and well with no nose bleeds, gastrointestinal bleeding or shortness of breath. He had been treated for testicular cancer on two occasions. No brain or spine AVMs were detected on MRI in father and son.

Primary lymphedema case

PL1 with EPHB4: c.[1230C>G];[=]; p.[Asn410Lys];[=]

PL1:II.3 was seen at the St. George's Hospital Primary Lymphoedema Clinic many years ago with left lower limb lymphedema onset 18 to 24 months of age. There was no history of fetal hydrops, congenital heart disease (CHD), CM, AVM or HHT. The right foot subsequently began to swell. On examination at the age of 14 years, she had bilateral foot and ankle lymphedema. The right was more swollen than the left, with a couple of small dysplastic nails, consistent with lymphedema. Lymphoscintigraphy imaging was consistent with primary lymphedema demonstrating abnormal drainage of tracer in both legs with evidence of deep rerouting via the popliteal lymph nodes, which is a recognised feature of primary lymphedema (Figure 2A). There was reduced uptake of tracer in the right and left ilioinguinal nodes after 2 hours.

Her maternal half-sister, PL1:II.2, did not complain of peripheral swelling but examination revealed a 'doughy' texture of the tissue in the feet. No prominent veins were seen. The lymphoscintigram (image not shown) demonstrated sluggish lymphatic drainage in both legs but without evidence of deep rerouting.

Her niece, PL1:III.1, was born following an uneventful pregnancy with no increased NT and no fetal hydrops. There was no edema at birth. She developed left foot lymphedema at the age of 2 years. Her lymphoscintigram showed abnormal drainage with reduced quantification in the left leg but without evidence of deep rerouting via the popliteal lymph nodes (Figure 2B). The drainage in the right leg appeared normal, however, in the 15-min scan (not shown) she had a significant early

drainage of tracer to the right thigh (but not the left). Therefore, the right lower limb 2-hour quantification figure is likely to be falsely elevated due to venous hypertension. No vein scan has been carried out to confirm this.

Supplementary Methods

EPHB4 somatic variant identification

To determine whether the c.2216G>A (chr7:100807483C>T, R739Q) variant in the proband (I.2) of the GLD_{UK} family is a somatic mosaicism or a germline variant, the ‘‘Somatic short variant discovery (SNVs + Indels)” pipeline of the GATK Best Practices Workflow (3) was utilised. Raw sequence reads for the sample were trimmed using Trimmomatic (4) and subsequently aligned to the GRCh37/hg19 human reference genome using the MEM algorithm of the Burrows–Wheeler Aligner (BWA) (5). Duplicated reads were marked, and base quality scores were recalibrated using GATK. MuTect2 (6) was then used to identify somatic variants in the sample, with a “panel of normals” generated from whole exome sequencing data of 31 randomly selected samples of the St George’s University Genetics Centre Exomes Inventory, which were pre-processed and aligned following the same procedure described above. A final VCF file with all the somatic variants and estimated allele-loads of the GLD_{UK}:I.2 sample, which passed the standard Mutect2 filters, was generated. Scripts used for the described analysis can be found online (Pre-processing/Alignment: https://github.com/sgul-genetics-centre-bioinformatics/Next-Generation-Sequencing-Pipelines; Unpaired Somatic Calling: https://github.com/digrigor/Unpaired_somatic_variant_calling).

Supplementary References

1. Martin-Almedina S, Martinez-Corral I, Holdhus R, Vicente A, Fotiou E, Lin S, et al. EPHB4 kinase-inactivating mutations cause autosomal dominant lymphatic-related hydrops fetalis. *Journal of Clinical Investigation.* 2016;126(8):3080-8.
2. Gershanik JJ, Jonsson HT, Jr., Riopel DA, and Packer RM. Dietary management of neonatal chylothorax. *Pediatrics.* 1974;53(3):400-3.
3. Van der Auwera GA, Carneiro MO, Hartl C, Poplin R, Del Angel G, Levy-Moonshine A, et al. From FastQ data to high confidence variant calls: the Genome Analysis Toolkit best practices pipeline. *Curr Protoc Bioinformatics.* 2013;43:11.0.1-.0.33.
4. Bolger AM, Lohse M, and Usadel B. Trimmomatic: a flexible trimmer for Illumina sequence data. *Bioinformatics.* 2014;30(15):2114-20.
5. Li H, and Durbin R. Fast and accurate long-read alignment with Burrows-Wheeler transform. *Bioinformatics.* 2010;26(5):589-95.
6. Benjamin D, Sato T, Cibulskis K, Getz G, Stewart C, and Lichtenstein L. Calling Somatic SNVs and Indels with Mutect2. *bioRxiv.* 2019:861054.
7. Yu J, Streicher JL, Medne L, Krantz ID, and Yan AC. EPHB4 Mutation Implicated in Capillary Malformation-Arteriovenous Malformation Syndrome: A Case Report. *Pediatr Dermatol.* 2017;34(5):e227-e30.

Supplementary Tables and Figures

Supplementary Figure 1: Pedigrees for lymphatic-related fetal hydrops (LRFH) cases.

Updated pedigrees of the two families originally published in Martin Almedina *et al.*, GLD_{UK} family (top) and GLD_{NOR} family (bottom) (1). Affected individuals are indicated with filled circles or squares. Genotypes indicate which samples have been sequenced and minus signs (-) represent the WT allele and plus signs (+) represent the mutant allele of the *EPHB4* gene. Triangles denote first trimester miscarriages. The arrow indicates the proband. A diagonal line through a square or circle indicates the individual has deceased.

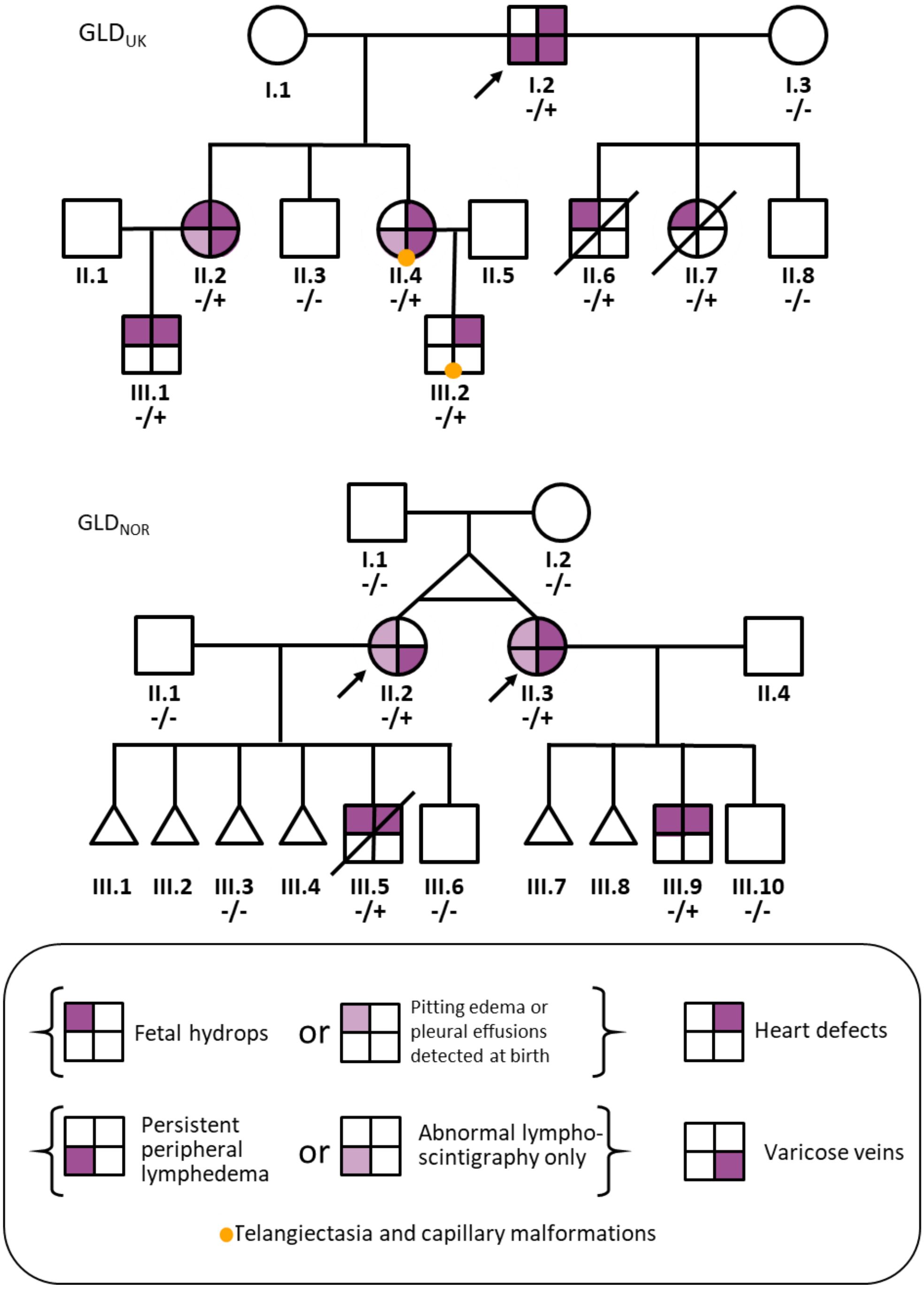

Supplementary Figure 2: Pedigrees for new non-immune fetal hydrops cases of unknown etiology. Pedigrees of the families FH1-FH5. Affected individuals are indicated with filled circles or squares. Arrows indicate the proband. Genotypes indicate which samples have been sequenced and minus signs (-) represent the WT allele and plus signs (+) represent the mutant allele of *EPHB4* gene. Triangle indicates first trimester miscarriages; diamonds indicate completed pregnancies beyond 20 weeks gestation and numbers within the triangle or diamond indicate the number of cases. A diagonal line through a square or circle indicates the individual has deceased. IUD, intrauterine death.

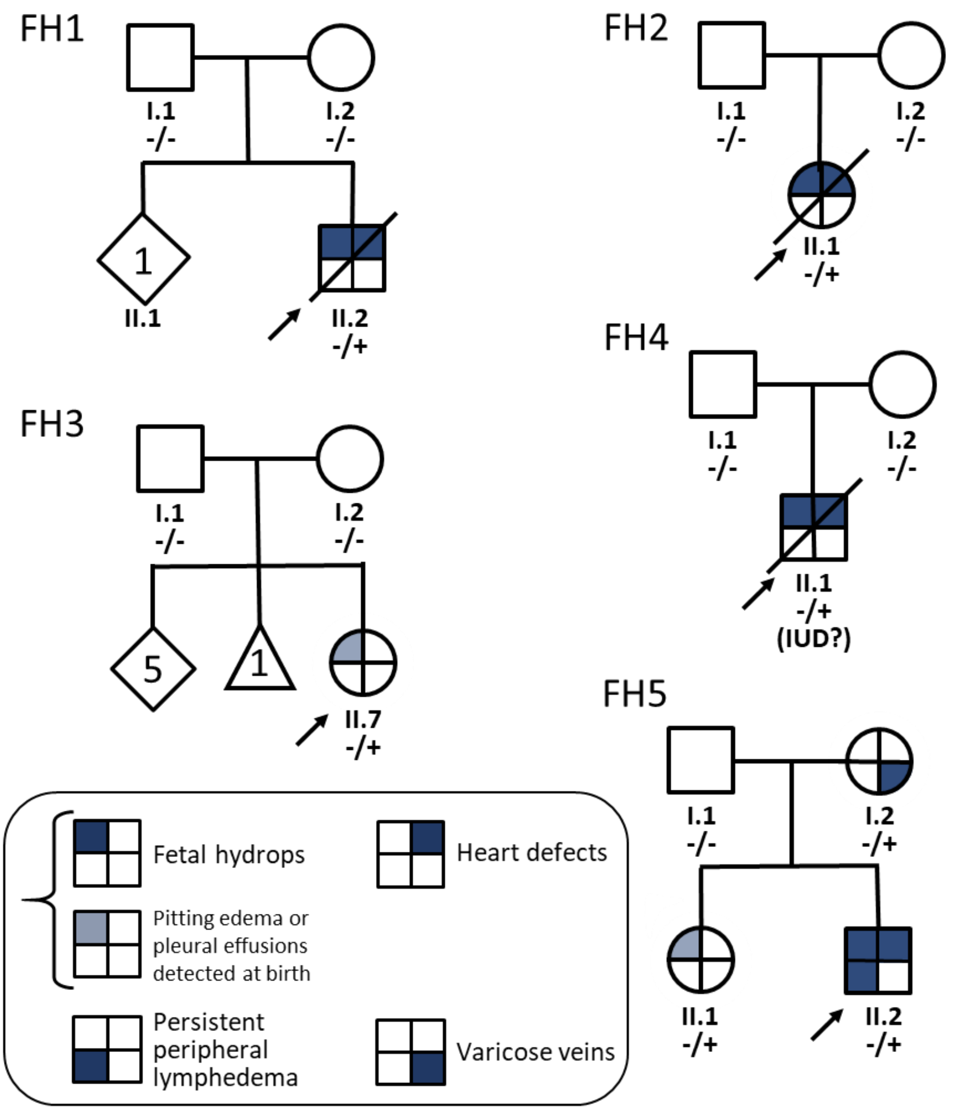

Supplementary Figure 3: Pedigrees for vascular anomaly cases and primary lymphedema cases. Pedigrees of the VA1 and VA2 families (top) and the PL1 family (bottom). Affected individuals are indicated with filled circles or squares. Arrows indicate the proband. Genotypes indicate which samples have been sequenced and minus signs (-) represent the WT allele and plus signs (+) represent the mutant allele of *EPHB4* gene.

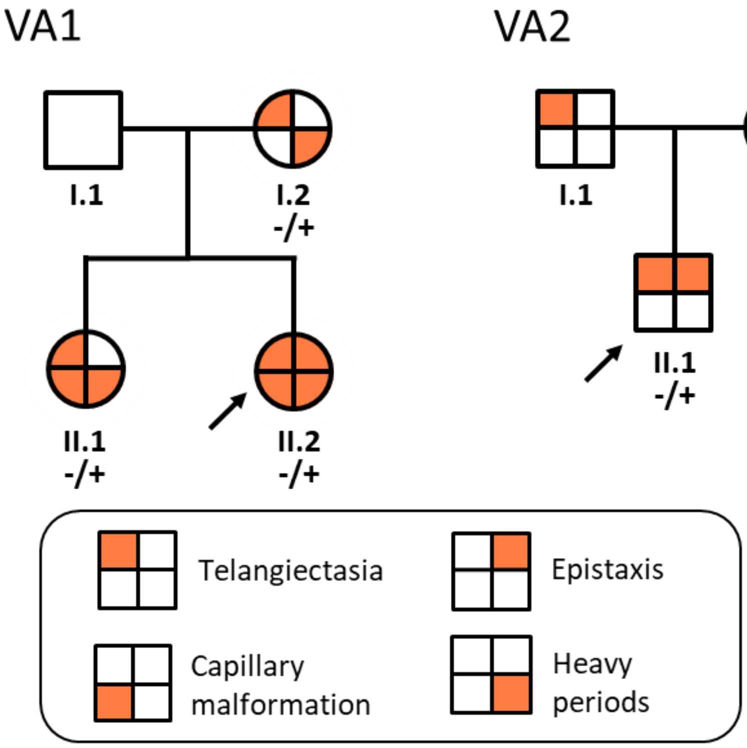

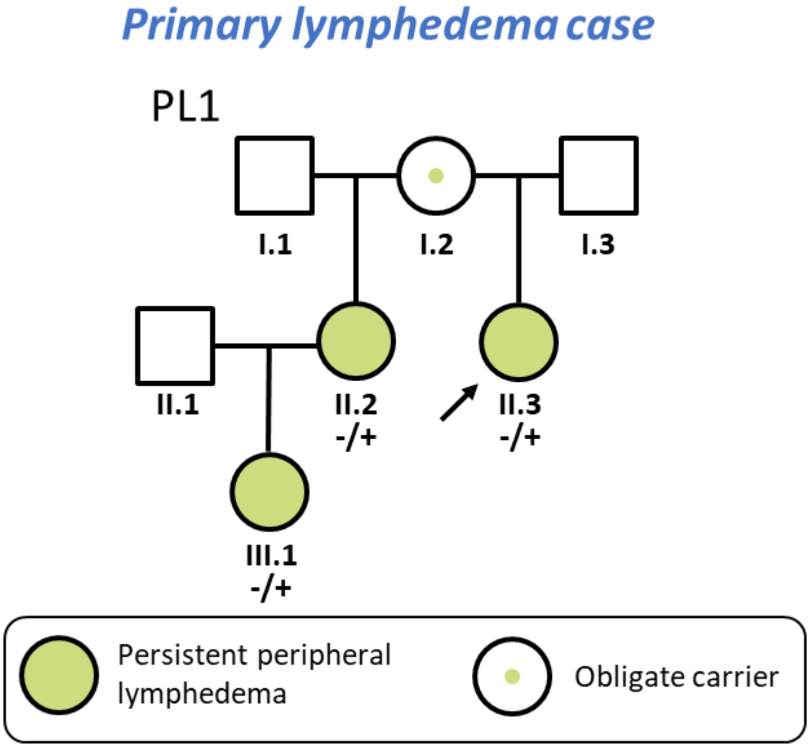

Supplementary Figure 4: Model showing the position of the 13 variants investigated along EPHB4 protein domains and functional regions.

Schematic representation of the WT EPHB4 protein (top) and the location of the 11 missense variants along the protein domains and functional regions, including the three control SNPs (grey) and the pD802G variant associated with CM-AVM2 (yellow) published by Yu *et al.* (7). Two variants are predicted to cause protein truncation. Schematic representation of the predicted mutated p.Q711X EPHB4 protein (middle) and p.S254Tfs*10 EPHB4 protein (bottom), resulting from a single nucleotide insertion at position 254 causing a frameshift and truncation of EPHB4 after 10 amino acids, are shown too. All variants are colour coded matching the corresponding pedigrees shown in Supplemental Figures 1-3. CM-AVM2, capillary malformation-arteriovenous malformation type 2; FH, fetal hydrops; LRFH, lymphatic-related fetal hydrops; PL, primary lymphedema; SNP, single nucleotide polymorphism; VA, vascular anomaly; LBD, ligand binding domain; SAM, sterile alpha motif.

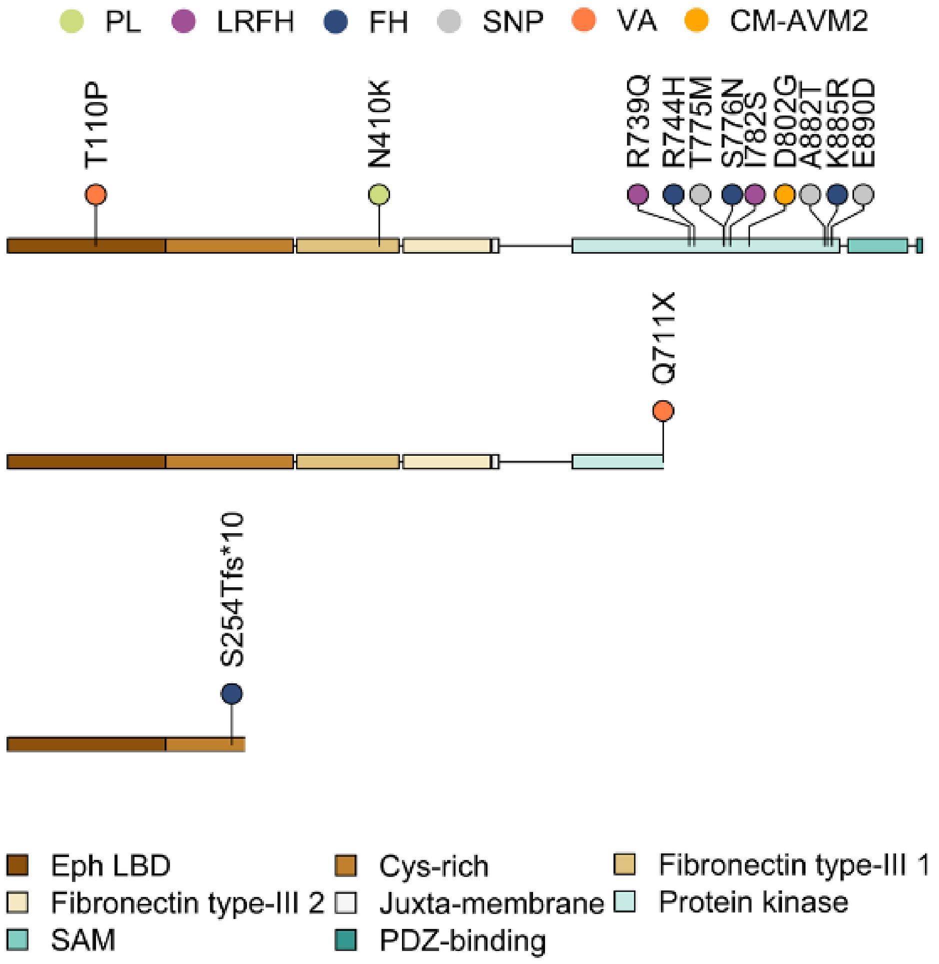

Supplementary Figure 5: EPHB4 protein sequences alignment showing conservation of residues of the 11 missense variants investigated.

(A) Sequences of EPHB4 proteins from *Homo sapiens* (Human), *Pan troglodytes* (chimpanzee), *Colobus angolensis* (Angola colobus), *Rhesus macaque* (rhesus), *Papio Anubis* (baboon), *Callithrix jacchus* (marmoset), *Mus musculus* (mouse) and *Canis lupus familiaris* (dog) were aligned using T-Coffee multiple sequence alignment. Asterisk (*) indicates the highly conserved residues. Red boxes show the positions of the amino acid substitutions for the potentially pathogenic variants investigated in this study. The positions of the three control SNPs are also indicated. **(B)** Sequences of different human tyrosine kinase receptors, containing the highly conserved HRD loop (blue box) were aligned. The locations of variants p.R739Q and p.R744H in the conserved region are indicated. All variants are colour coded matching the corresponding pedigrees shown in Supplemental Figures 1-3. Abbreviations are the same as in Supplementary Figure 4.

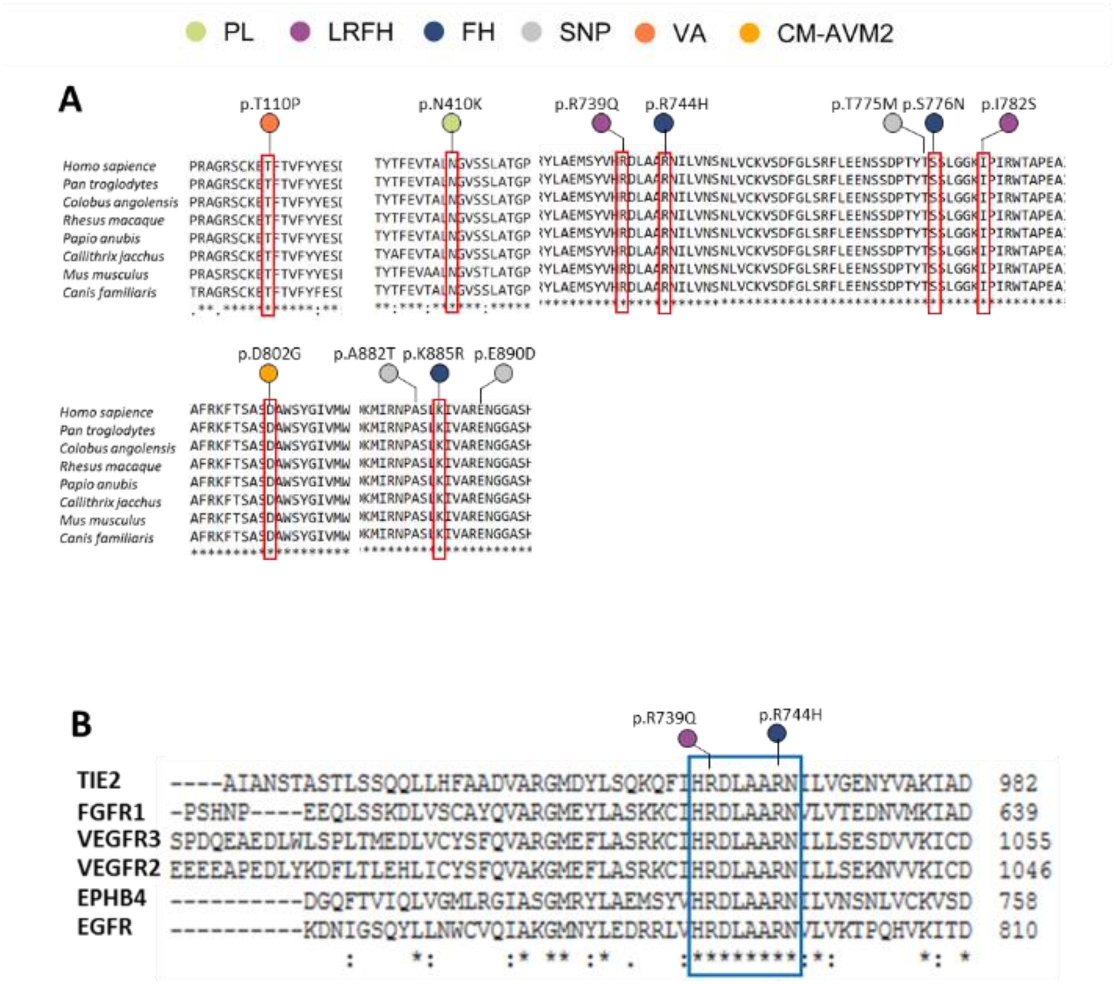

Supplementary Figure 6: Mosaicism in GLD_{UK}:I.2.

(A) Sanger sequencing trace confirming the *EPHB4* c.2216G>A variant in the reverse transcript of GLD_{UK}:I.2. **(B)** Visualisation of the aligned exome sequencing reads in IGV covering the c.2216G>A variant for GLD_{UK}:I.2 shows 63% WT C and 37% mutant T suggesting mosaicism. **(C)** Somatic calling metrics suggest that GLD_{UK}:I.2 is most likely mosaic for this variant.

DP, Approximate read depth; ECNT, Number of events in this haplotype; POP_AF, population allele frequencies of alternative alleles; P_CONTAM, Posterior probability for alternative allele to be due to contamination; P_GERMLINE, Posterior probability for alternative allele to be germline variants; TLOD, Tumor LOD score; GT, Genotype; AD, Allelic depths for the reference and alternative alleles in the order listed; AF, Allele fractions of alternative alleles in the tumor; F1R2, Count of reads in F1R2 pair orientation supporting each allele; F2R1, Count of reads in F2R1 pair orientation supporting each allele; MBQ, median base quality; MFRL, median fragment length; MMQ, median mapping quality; MPOS, median distance from end of read; SA_MAP_AF, MAP estimates of allele fraction given z; SA_POST_PROB, posterior probabilities of the presence of strand artifact.

Supplementary Figure 6

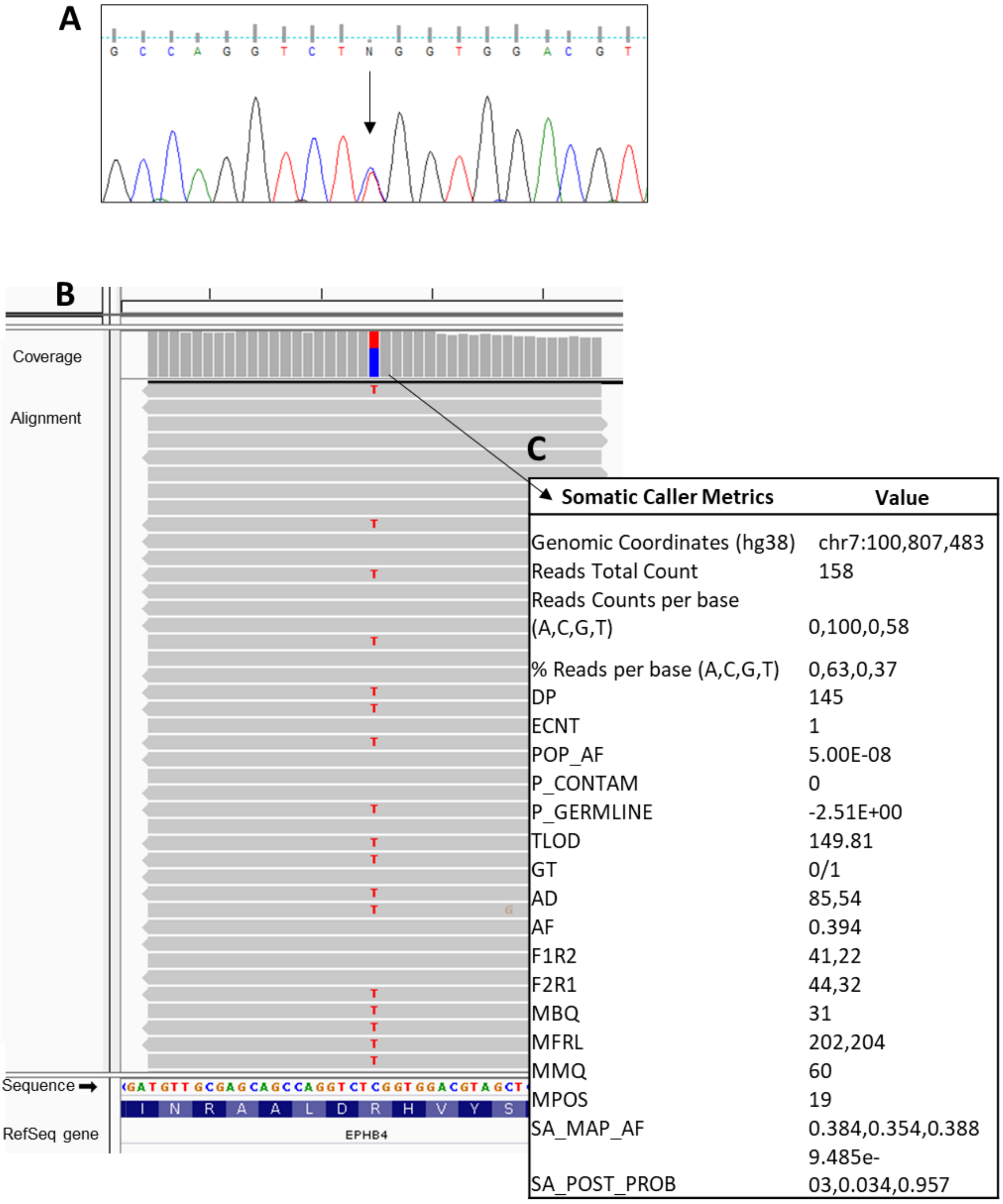

Somatic Caller Metrics	Value
Genomic Coordinates (hg38)	chr7:100,807,483
Reads Total Count	158
Reads Counts per base (A,C,G,T)	0,100,0,58
% Reads per base (A,C,G,T)	0,63,0,37
DP	145
ECNT	1
POP_AF	5.00E-08
P_CONTAM	0
P_GERMLINE	-2.51E+00
TLOD	149.81
GT	0/1
AD	85,54
AF	0.394
F1R2	41,22
F2R1	44,32
MBQ	31
MFRL	202,204
MMQ	60
MPOS	19
SA_MAP_AF	0.384,0.354,0.388
SA_POST_PROB	9.485e-03,0.034,0.957

Supplementary Figure 7: Full unedited western blots for Figure 3.

Original western blots (one representative of three experiments is shown) with black outline indicating which sections have been used in Figure 3A and B in the paper (See legend to Figure 3 for interpretation). The first lane on the left of each blot corresponds to the molecular weight markers in kDa.

Supplementary Figure 7

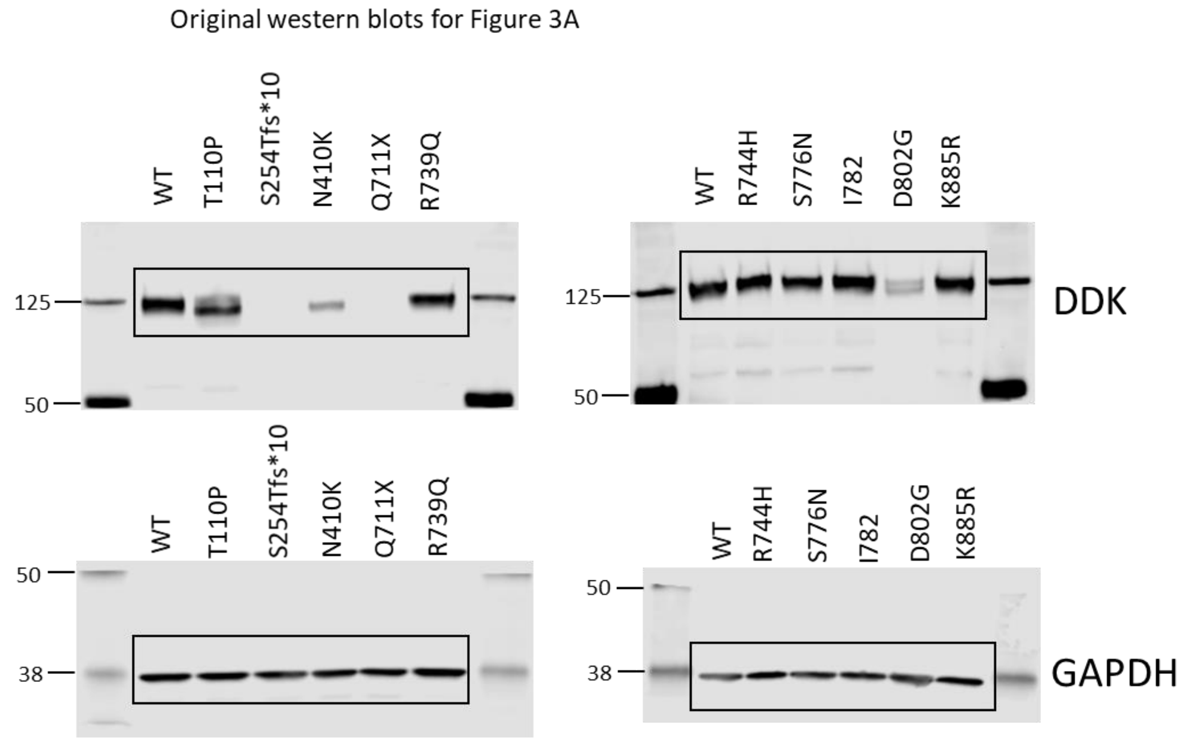

Supplementary Figure 7

Original western blots for Figure 3B

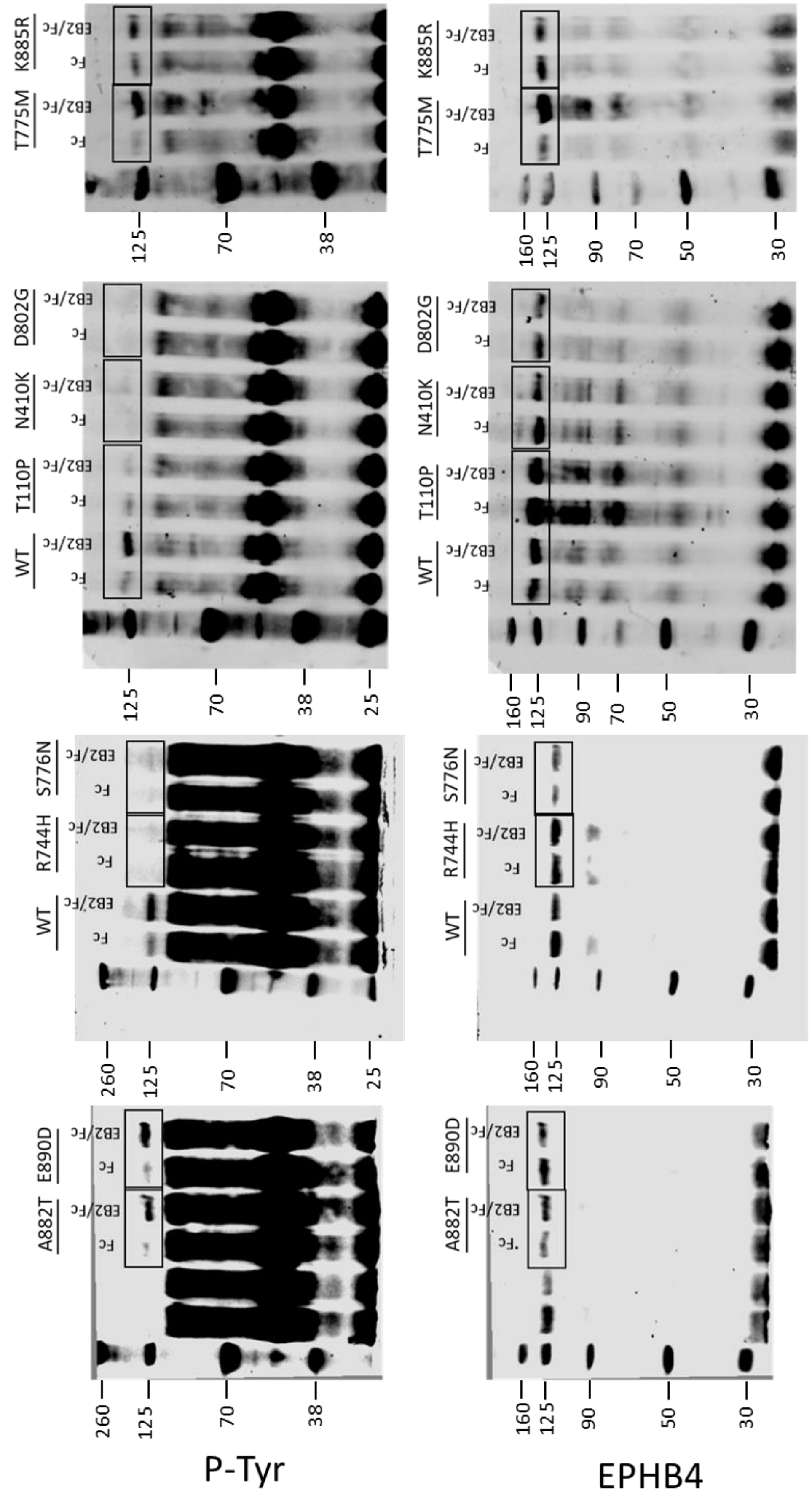

Supplementary Table 1: Full clinical information.

Complete clinical information associated with the phenotype of the eight new index cases and affected family members with an *EPHB4* variant of uncertain significance (FH1-FH5, VA1-VA2 and PL1) included in this study. The *EPHB4* index cases and affected family members previously reported by Martin-Almedina et al. (GLD_{UK} and GLD_{NOR}) have been updated and included for comparison (1). Refer to the Supplementary Information for detailed clinical description of each case.

*, monozygotic twins; ‡, only listed if clinically assessed for telangiectasia. As, Ascites; ASD, atrial septal defect; CT, chylothoraces; F, female; FH, fetal hydrops; GLD, generalized lymphatic dysplasia; IUD, intrauterine death; L, Left; LD, Learning difficulties; M, male; ND, neonatal death; NT, Nuchal translucency scan; PC, pericardial effusions; PE, pleural effusions; PDA, patent ductus arteriosus; PFO, patent foramen ovale; PL, primary lymphedema; spont, spontaneously; R, Right; SC, subcutaneous edema; TC, thoracocentesis in utero; US, ultrasound; VA, vascular anomaly; VSD, ventricular septal defect ; blank fields indicate the information was either not recorded or not applicable. In the persistent peripheral lymphedema column, () indicates the lymphatic abnormality was only observed on lymphoscintigraphy.

Supplementary Table 2: Oligonucleotides (5’-3’) used for site-directed mutagenesis of human EPHB4.

Substituted nucleotides are highlighted in bold and underlined. All constructs were verified by Sanger sequencing.

c.328A>C (p.Thr110Pro)	Forward	GAAGACGGTGAAGG**G**CTCCTTGCAGGAGC
	Reverse	GCTCCTGCAAGGAG**C**CCTTCACCGTCTTC
c.760_761insC (p.Ser254Thrfs*10)	Forward	CGGAGCACAGC**G**TGCAGCCCGTGAC
	Reverse	GTCACGGGCTGCA**C**GCTGTGCTCCG
c.1230C>G (p.Asn410Lys)	Forward	GAGGATACCCC**C**TTCAATGCAGTGACCTCAAAG
	Reverse	CTTTGAGGTCACTGCATTGAA**G**GGGGTATCCTC
c.1990G>A (p.Glu664Lys)	Forward	CCATGATGGAGGCCT**T**GCTCAGAAACTCACG
	Reverse	CGTGAGTTTCTGAGC**A**AGGCCTCCATCATGG
c.2131C>T (p.Gln711X)	Forward	GGATGACTGTGAACT**A**TCCG**T**CGTTTAGCCGCAG
	Reverse	CTGCGGCTAAACGACGGA**T**AGTTCACAGTCATCC
c.2231G>A (p.Arg744His)	Forward	CGAGACCTGGCTGCTC**A**CAACATCCTAGTCAAC
	Reverse	GTTGACTAGGATGTTG**T**GAGCAGCCAGGTCTCG
c.2324C>T (p.Thr775Met)	Forward	CTCCCAGGGAGCTC**A**TGTAGGTGGGATCG
	Reverse	CGATCCCACCTACA**T**GAGCTCCCTGGGAG
c.2327G>A (p.Ser776Asn)	Forward	CCCACCTACACGA**A**CTCCCTGGGAGGA
	Reverse	TCCTCCCAGGGAG**T**TCGTGTAGGTGGG
c.2405A>G (p.Asp802Gly)	Forward	CACTTCCGCCAGTG**G**TGCCTGGAGTTACG
	Reverse	CGTAACTCCAGGCA**C**CACTGGCGGAAGTG
c.2590C>T (p.Arg864Try)	Forward	GGAAGCGGGGCC**A**GGCATTCCGGTC
	Reverse	GACCGGAATGCC**T**GGCCCCGCTTCC
c.2644G>A (p.Ala882Thr)	Forward	ATGATCCGGAACCCC**A**CCAGCCTCAAAATCG
	Reverse	CGATTTTGAGGCTGG**T**GGGGTTCCGGATCAT
c.2654A>G (p.Lys885Arg)	Forward	CGGGCCACGATT**C**TGAGGCTGGCGG
	Reverse	CCGCCAGCCTCA**G**AATCGTGGCCCG
c.2670G>T (p.Glu890Asp)	Forward	GTGGCCCGGGA**T**AATGGCGGGGC
	Reverse	GCCCCGCCATT**A**TCCCGGGCCAC
c.2767A>G (p.Arg923Gly)	Forward	CGAAACTTTCTTCGTATC**C**TCCCATTTTGATGGCCCG
	Reverse	CGGGCCATCAAAATGGGA**G**GATACGAAGAAAGTTTCG

Supplementary Table 3: Summary of the results from the functional analysis of *EPHB4* variants investigated in this study.

Protein changes and domains affected for each variant are described in the table. Protein expression, protein activity (P-Tyr) and subcellular localization by immunofluorescence (IF) staining findings are summarised together with the proposed effect of variant based on our results.

ID	Predicted protein change	Domain affected	Protein Expression	Protein Activity (P-Tyr)	Subcellular localization by IF staining	Proposed effect of variant based on our test results
FH5	K885R	TKD	Normal	Normal	Membrane	Benign?
GLD$_{UK}$	R739Q	TKD	Normal	Reduced	Membrane	Pathogenic
FH1, FH2	R744H	TKD	Normal	Reduced	Membrane	Pathogenic
FH3	S776N	TKD	Normal	Reduced	Membrane	Pathogenic
GLD$_{NOR}$	I782S	TKD	Normal	Reduced	Membrane	Pathogenic
FH4	S254Tfs*10	-	Loss of protein	-	-	Pathogenic
VA1	Q711X	-	Loss of protein	-	-	Pathogenic
VA2	T110P	LBD	Reduced	Reduced	ER? + cellular aggregates	Pathogenic
CM-AVM2	D802G	TKD	Reduced	Reduced	ER? + cellular aggregates	Pathogenic
PL1	N410K	FND	Reduced	Reduced	ER? + cellular aggregates	Pathogenic

CM-AVM, capillary malformation-arteriovenous malformation; ER, endoplasmic reticulum; FH, fetal hydrops; FND, fibronectin domain; GLD, generalized lymphatic dysplasia; LBD, ligand binding domain; PL, primary lymphedema; TKD, tyrosine kinase domain; VA, vascular anomaly.
